# Riems influenza a typing array (RITA): An RT-qPCR-based low density array for subtyping avian and mammalian influenza a viruses

**DOI:** 10.1038/srep27211

**Published:** 2016-06-03

**Authors:** Bernd Hoffmann, Donata Hoffmann, Dinah Henritzi, Martin Beer, Timm C. Harder

**Affiliations:** 1 Institute of Diagnostic Virology, Friedrich-Loeffler-Institut, Suedufer 10, 17493 Greifswald-Insel Riems, Germany

## Abstract

Rapid and sensitive diagnostic approaches are of the utmost importance for the detection of humans and animals infected by specific influenza virus subtype(s). Cascade-like diagnostics starting with the use of pan-influenza assays and subsequent subtyping devices are normally used. Here, we demonstrated a novel low density array combining 32 TaqMan^®^ real-time RT-PCR systems in parallel for the specific detection of the haemagglutinin (HA) and neuraminidase (NA) subtypes of avian and porcine hosts. The sensitivity of the newly developed system was compared with that of the pan-influenza assay, and the specificity of all RT-qPCRs was examined using a broad panel of 404 different influenza A virus isolates representing 45 different subtypes. Furthermore, we analysed the performance of the RT-qPCR assays with diagnostic samples obtained from wild birds and swine. Due to the open format of the array, adaptations to detect newly emerging influenza A virus strains can easily be integrated. The RITA array represents a competitive, fast and sensitive subtyping tool that requires neither new machinery nor additional training of staff in a lab where RT-qPCR is already established.

Influenza A viruses (IAVs) cause infections in various avian and several mammalian species, including humans^1^. The genomes of IAVs are composed of eight negative sensed single stranded RNA segments^2^. Due to the segmented nature of the genome combinatorial exchanges of genome segments, so-called reassortments may occur when different IAVs replicate in the same host cell. This phenomenon significantly increases IAV genotypic variability and may also contribute to ad hoc shifts in viral phenotypes. Two glycoprotein species located in the viral membranes of IAVs, the haemagglutinin (HAs) and the neuraminidases (NAs), represent the major antigenic determinants that define the subtype of the virus^2^. Reassortment events that changed a combination of HA and NA glycoproteins and created a novel antigenic phenotype in an immunologically naïve human population have been the basis of pandemic, i.e., global waves of human IAV infections^3^. Only a subset of all known IAV subtypes infect mammalian species, whereas avian metapopulations perpetuate the full spectrum of 16 different HA and 9 NA entities^4^,^5^,^6^. The only exceptions are the recently described bat influenza viruses H17N10 and H18N11 that were up to now detected in bats only^7^,^8^.

The symptoms, if any, of IAV induced human disease vary from mild to severe respiratory disorders. However, rarely occurring severe systemic disorders with substantial fatality rates have been reported, as well^9^. In temperate climates, there is a sharply defined human influenza season, during which a total of 1 billion people are affected annually^10^. Influenza A-related diseases induced in susceptible livestock, particularly poultry and swine, are highly variable and may have economic implications due to animal losses, reduced production rates and/or trade restrictions. The severity of influenza infections in animals is, to some extent, related to the species, age and constitution of the host, but there are also influential viral determinants of pathogenicity. This is particularly true for highly pathogenic forms of avian influenza (AI) caused by certain lineages of the AIV subtypes H5 and H7. Avian infections with either of these subtypes, independent of their pathogenicity, are notifiable and call upon rigorous restriction and eradication measures (e.g. “stamping out”)^11^.

Due to the potentially far-reaching consequences of the detection of certain IAV subtypes, a rapid and reliable diagnostic subtyping assay is of paramount importance. Virus subtyping directly from original sample material would considerably reduce the time until diagnosis. Here, we describe a very broad molecular subtyping assay system based on reverse transcription quantitative PCR (RT-qPCR) in a multiwell layout that is easily prepared and runs on standard lab equipment. By using a hydrolysis probe technique (TaqMan^®^), one of the most powerful and widespread methodologies in diagnostic microbiology^12^ was applied. The format is referred to as the Riems Influenza A Typing Array, abbreviated RITA. The RITA assay is the first system that combines simple duplex TaqMan^®^ reactions into one diagnostic tool, without the need for further equipment for detection or identification of 14 HA and 9 NA subtypes of influenza A viruses. More than 400 IAV isolates and clinical material from 63 swab samples were successfully subtyped to qualify this protocol as eminently suitable for routine procedures.

## Materials and Methods

### Viruses

Samples from 404 influenza viral strains representing 16 HA and 9 NA subtypes pre-typed (either serologically or by sequencing) at the National Reference Laboratory for Avian Influenza (NRL-AI, FLI) were used for analytical validation of the newly developed assays. These isolates are referred to as the “validation panel” throughout this manuscript. Tables 1 and 2 provide a condensed overview of the subtypes and species of origin, and supplemental Table S1 (available online) details the individual viral strains. Furthermore, to assess the applicability of RITA to investigate field samples containing various loads of IAV RNA, 62 swabs from wild birds (originating from wild bird monitoring) and swine (diagnostic samples from diseased swine herds) were tested. Sanger sequencing of amplicons generated from the field sample RNAs by pan-HA and pan NA RT-PCR-protocols^13^,^14^ (Henritzi *et al.* under review) was used to verify the RITA subtyping results.

### Primers and probes

The pan-influenza A IAV-M1.2 assay^15^ was implemented in RITA as a control for the presence of IAV RNA in the sample tested and to provide quantification cycle values (Cq) for comparison to other HA- and NA-specific RT-qPCRs in the array. By *in silico* analyses of publicly available IAV sequences, primers and probes specific for the different HA and NA subtypes were identified. The primer and probe sets used in this study are detailed in Table 3. Oligos were synthesized by Metabion GmbH (Martinsried, Germany) and stored at −20 °C until use.

### RNA extraction

Viral RNA was extracted from supernatants of infected MDCK cell cultures or allantoic fluids of embryonated chicken eggs using the MagNA Pure LC Total Nucleic Acid Isolation Kit (Roche, Penzberg, Germany), according to the manufacturer’s instructions, on a MagNA Pure LC 2.0 system (Roche). Clinical material (swab samples) was extracted manually using the Qiagen Viral RNA kit (Qiagen, Hilden, Germany) or by the Qiagen MagAttract Kit operated on a KingFisher Biosprint96 device (Qiagen).

### Individual RT-qPCRs and plate arrangement

All individual RT-qPCRs were performed on the Bio-Rad CFX 96 real-time PCR (Bio-Rad, Munich, Germany) platform in a 96-well format using the AgPath-ID One-Step kit (Applied Biosystems, Foster City, CA). Using a heterologous internal control system^16^, the integrity of the reagents was assured, and inhibition phenomena were excluded. The composition of a single reaction of 12.5 μl was as follows: 1 μl of RNase-free water, 6.25 μl of 2x RT-PCR buffer, 0.5 μl of RT-PCR Enzyme Mix, 0.25 μl of internal control template, 1 μl of primer-probe-mix for the internal control and 1 μl of IAV specific primer-probe-mix. Finally, 2.5 μl of RNA template was added. In its current lay-out, 32 wells (i.e., four eight-well stripes) of a 96 well PCR plate are used per RITA analysis. Thus, running three samples in parallel on one 96-well plate is possible. A typical lay-out of the 32 wells is depicted in supplemental Fig. S1, available online. During the setup of a plate, RNase-free water (0.5 μl), the IAV specific primer-probe-mix (1 μl) and the primer-probe-mix for the internal control (1 μl) were added to each individual well. At this stage of preparation, storage of the plate at −20 °C is possible, and bulk production of plates is advisable. After thawing the required amount of plates, the master mixes for each sample were prepared, which were composed of the RT-PCR buffer, the enzyme mix, the internal control RNA, residual water and the extracted viral RNA. Ten microliters of this mixture was added to each of the 32 wells of the test using a multi-channel pipette. Thus, a total of 80 μl of extracted RNA is required per sample to feed the 32 wells for a complete characterization. For PCR amplification, the following temperature profile was applied: 10 min at 45 °C (reverse transcription) and 10 min at 95 °C (inactivation of the reverse transcriptase/activation Taq polymerase), followed by 45 cycles of 15 sec at 95 °C (denaturation), 20 sec at 56 °C (annealing) and 30 sec at 72 °C (elongation). Fluorescence values (FAM, HEX) were collected during the annealing step.

In order to confirm the integrity of all of the target- and internal control-specific primer-probe-mixtures a positive control containing a mixture of RNA of all H and N subtypes targeted (panIAV-PC) is recommended. The panIAV-PC is a mixture of IAV-RNA from all IAV subtypes analysed in the array. However, based on the internal control system and the inclusion of the pan-influenza A IAV-M1.2 assay^15^, the application of RITA without the co-analysis of panIAV-PC is also valid. The analysis of RNase free water as a no template control (NTC) may identify cross-contamination of the master mix. Analysis of the NTC and the panIAV-PC was required after a new batch of arrays was produced, or the arrays were stored in the freezer for a prolonged period of time (more than 4 weeks) before use.

## Results

### RT-qPCRs and construction of a 32-well PCR array

By *in silico* analysis of published sequence data, primer and probes for the generic detection of HA subtypes H1 to H13 and H16 and all 9 NA subtypes were selected with the aim of detecting the broadest possible spectrum within a given subtype. To this end, various RT-qPCRs were newly developed, and their performance, analysed. In addition, published RT-qPCR subtyping protocols were evaluated, as well. Finally, the best performing assays were combined into the low density array referred to as “Riems Influenza A Typing Array” (RITA). The final makeup of RITA consisted of single assay detection of subtypes H4, H6, H8, H9, H11, H12, H13 and H16; two assays for H1, H2, H3, H5 and H10 detection; and three assays for H7 detection. NA subtyping was performed by single assays, except for the N3 subtype, which required two assays. This process resulted in the ultimate 32 well format RITA, which was evaluated using a selected set of 404 IAV isolates (detailed in supplemental Table S1). All samples were also run in the pan-influenza IAV-M1.2-assay^15^ included in the 32 well RITA to verify the presence of IAV RNA in general and to obtain an estimation of the viral genome load of each individual sample by generic amplification of a highly conserved region of genome segment 7 of all IAV isolates^15^.

### Analytical sensitivity of RT-qPCRs

The analytical sensitivity of each single assay of RITA was evaluated using a dilution series of matching viral RNAs and compared with the Cq-values of the respective RNA obtained by the generic-influenza IAV-M1.2 test. The analyses showed that all subtype specific assays were similar sensitive as the generic-influenza assay. At the maximum 10-fold less viral RNA was detected, using the serotype specific assays compared to the generic pan-influenza test. In some cases, a better analytical sensitivity for the serotype specific assay was measured (data available upon request).

### Analytical specificity of RITA

Analytical specificity was assessed by the determination of inclusivity and exclusivity on a validation panel of 404 different IAV isolates. The results are summarized in Table 4. As an example, “H1-subtype mix 3” detected 51 out of 54 H1 virus isolates correctly, whereas “H1 mix 27” detected 53 out of 54 H1 RNAs. Isolates that were not detected differed between the two assays: “H1 mix 3” did not detect A/mallard/Germany/R3036/2007 (H1N1), A/swine/Germany-NI/R369/2009 (H1N2) or A/swine/Germany/AR1372/2015, whereas “H1 mix 27” failed to detect A/England/1/1951 (H1N1). Therefore, with a combination of both H1 mixes, all H1 isolates represented in the validation panel were correctly identified as representatives of the H1-subtype. While “H1 mix 3” proved to be exclusively specific for H1, “H1-mix 27” showed a weak cross-reactivity with 37 H6 isolates and three isolates of the H8 subtype and therefore had a specificity of only 89.9%. However, it should be noted that there was a difference in the Cq-values (Δ C_q_) between the true specific and the false positives of more than 10 units when comparing the pan IAV-M1.2 assay to the false positive signals, and 9 units when comparing the specific assay to the false positive reacting assay results.

Both H2-detecting assays were specific for subtype H2 and identified 18 out of 18 H2 isolates correctly, and an unspecific cross-reactivity was recorded for only one of the H5 isolates for the “IAV-H2-Mix 10” (Δ C_q_ > 10 generic assay, Δ C_q_ > 8 specific assays; Table 4). Furthermore, from a total of 40 H3 isolates of the validation panel, 35 were detected by “IAV-H3- Mix 3”, and 40 of 40 scored positive with the “IAV-H3-Mix 14” (Table 4). Additionally, all 17 H4 isolates were correctly identified by “IAV-H4-Mix 15” (Table 4). Specific detection of all H5 sequences was achieved by “IAV-H5a-Mix 1” (88/88) and also approximated by the “IAV-H5-Mix 1” assay (87/88). Subtype H6 sequences (in a total of 61 isolates) were all correctly evaluated by the “IAV-H6-Mix 8” assay (Table 4). Interestingly, for the complete identification of the H7 subtype sequences, three assays had to be implemented, providing the following coverage: “IAV-H7-CODA” detected 33 out of 35 correctly, with cross-reactivity with an H5 isolate, an H10 isolate and an H15 isolate; IAV-H7-Mix 2 detected 35 out of 35 correctly, with cross-reactions with six of the H10 isolates; “IAV-H7-Mix 2.2” detected all 35 isolates correctly. The nonspecific reactions again showed, in all cases, a Δ C_q_ > 10 compared to the specific reactions. Finally, the assays designed to detect the H8, H9, H11, H12, H13 and H16 subtypes reacted with the respective isolates and showed no cross-reactivity with any other subtypes. Nevertheless, both H10 assays exhibited nonspecific reactions in addition to the exact identification of all H10 subtypes: “IAV-H10-Mix 5” scored positive with 30 out of 40 H3 isolates with Cq-values that were at least three units higher than the values of the “IAV-M1.2” assay and the specific assay; “IAV-H10-Mix 9” scored positive with 30 out of 35 H7 isolates with a Δ Cq value of 5 compared to the IAV-M1.2 assay and Δ Cq value of >4 compared to the specific assay. However, with the redundancy of the two tests, all H10 isolates could be correctly identified.

The reactivity of the NA-subtype specific assays showed a 100% match to the applied isolates, with no cross-reactivity (Table 5).

### Diagnostic performance of RITA

The evaluation of diagnostic sensitivity was conducted using RNA from swab samples of wild birds or pigs. In general, subtyping was successful for those samples that exhibited Cq-values <34 with the pan IAV M1.2 assay. Out of 45 swab samples originating from wild aquatic birds, 45 samples exhibited (at least one) HA result and 44 samples (at least one) NA results by using RITA (Table 6). In 16 samples, additional reactivity with further subtypes was evident. Eleven of these additional reactivities apparently paralleled those already known from the validation panel; thus, no mixed IV infections were assumed for these samples. In contrast, mixed influenza infection was suspected in four samples because similar Cq-values for two or more HA and NA subtypes were obtained from multiple subtype assays. Verification of subtypes identified by RITA was achieved for the majority of bird samples by Sanger sequencing technology. However, samples showing Cq-values >30 in the generic IAV M1.2 assay did not regularly yield specific amplicons, especially for the HA, that were suitable for Sanger sequencing. The individual reaction patterns of all diagnostic samples are summarized in Table S2. Nasal swabs from swine were also used for subtype detection. For 15 out of 17 IAV positive samples, a subtype was unambiguously assignable by RITA (Table 6). One sample showed additional (cross-) reactivity, as previously seen from the validation panel. Specific subtypes identified by RITA were confirmed by subsequent Sanger sequencing (except for one sample for which no NA amplicon was obtained; Table S2). For one nasal swab sample, a mixed infection with more than one subtype was suspected (Table 6).

## Discussion

The objective of this study was to develop and validate an RT-qPCR based subtyping tool for rapid and reliable direct subtyping of influenza A viruses from original sample material of avian and mammalian hosts. The finally validated 32-well RT-qPCR array, named RITA, allows the differentiation of 14 HA and 9 NA subtypes, as well as generic IAV RNA detection and an internal control system. The rare subtypes H14 and H15 were not implemented in this RITA version due to their minor relevance and to maintain an easy to manage format (e.g. using multiples of eight).

RT-qPCR-based nucleic acid detection techniques are routinely implemented throughout diagnostic laboratories, and the RITA protocol is easily established in such laboratories. The single tube duplex assay structure of RITA provides very high sensitivity for the individual assay, as well as a very good handling versatility, because all 32 wells can be pipetted using a multiwell pipette and a single master mix per sample. This format also offers complete flexibility to modify existing or implement new assays targeting specific clades, HA cleavage sites (pathotyping for H5 and H7 subtypes;)^17^, new geographical variants or assays for the identification of relevant pathogens of differential diagnostic importance. In the light of this flexibility, RITA can easily be adapted to the effects of evolutionary drift that may lead to IAV variants not only escaping vaccine-induced immunity but also routine diagnostics^18^. The current plate design resembling a low density PCR array format allows analysis of three samples in parallel per 96 well plate. The plates can be prepared in advance and stored at −20 °C. A storage time of nine months did not lead to a loss of sensitivity. Using plates from the freezer, the overall setup and run time of RITA was less than three hours.

Recently, Elizalde and colleagues described a molecular subtyping approach for HA subtyping of IAV^19^, and the use of locked nucleic acids (LNA) within the probe sequence in this method rendered the RT-qPCR assays highly subtype-specific^19^. LNA probes are specific to such an extent that single nucleotide polymorphism (SNP) genotyping is possible^20^. However, with this implement, mismatches within the LNA probe binding region are less well tolerated, and the likelihood of false negative reactions increases. In addition, LNA-containing probes are more expensive than Taqman^®^ or minor groove binding (MGB^®^) probes. Using SYBR green as a non-specific detection tool, Tsukamoto and colleagues developed another RT-qPCR-based subtyping protocol for HA and NA of IAVs^21^. This approach was successfully applied to identify NA subtypes from wild bird samples originating from a surveillance program^22^. However, SYBR green-based detection is less specific than probe-based detection technologies because the fluorescent substance binds to any double-stranded DNA molecule amplified during PCR.

With RITA, we attempted to find a perfect balance between highly sensitive broad reactivity and extreme subtype specificity with the use of Taqman^®^/MGB-based probes and, if necessary, multiple tests per subtype. Certain performance traits of single RT-qPCRs implemented in RITA were already demonstrated in citations^15^,^16^,^23^,^24^,^25^,^26^; in addition, some newly designed assays were compared with published protocols^27^, produced superior results, and, hence, were included in the RITA format. However, due to the wide-stretched spectrum of the virus panel used to validate RITA, comprising more than 400 isolates, comparative analysis of every newly designed assay against published Taqman assays was not feasible. The current version of RITA is certainly aimed at a best fit to Eurasian IAVs. Yet, the flexible basis of the array system allows the adaptation/inclusion/substitution of single RT-qPCRs to create RITA formats that serve specific needs e.g. according to geography (Eurasian versus American genotypes) or epidemiology (outbreak-adapted assays) or differential diagnostics.

RITA was validated using 404 influenza A virus isolates from both avian and mammalian hosts, and unequivocal subtype identification was completely successful on virus isolates. The need to design primers and probes that guaranteed the broadest within-subtype reactivity and the inherent prerequisite of RITA to apply the same cycling conditions to all 32 assays implemented produced few trade-off effects with respect to specificity for some of the assays. Slight tendencies for the co-amplification of non-targeted HA subtypes were observed for some of the selected RT-qPCRs. However, these false amplicons were usually detected with a much lower sensitivity and thus could easily be identified as false-positives when comparing the true positive subtype-specific reactions to the Cq-values obtained in the pan IAV M1.2 assay. Nevertheless, this propensity of the RITA assays reduces only the reliability to detect minor virus species (particularly H1, H10) in mixed IAV infections. The necessity of discerning co-amplification from mixed infection became apparent when examining clinical material from swine and avian species. The mixed infection with subtypes H1 and N1 and N2 detected in one porcine nasal swab demonstrated the extraordinary capacity of RITA to identify mixed infections in one sample. Sample materials originating from aquatic wild birds also gave evidence of mixed infections by diverse influenza A virus subtypes when examined by RITA (Table 6). Mixed infections are a precondition of genome reassortment^28^, but discerning mixed infections by standard Sanger sequencing technologies is seriously hampered^29^. The current RITA protocol in principal allows the identification of mixed IAV infections, although the interpretation of data may require certain expertise and experience to distinguish true co-infections with different IAV subtypes from unintended co-amplification or sample contamination. For instance, samples that exhibit similar Cq values for two or more HA and NA subtypes likely represent true co-infections. In contrast, if a single major subtype is identified and one or more very minor subtypes are present in the sample, no clear decision is possible unless other subtyping methods (virus isolation, next-generation sequencing) produce corroborating results. Therefore, and to keep in touch with the evolution and geographic dissemination of IAVs, the single RITA assays will have to be reassessed and improved on a regular basis.

Rapid and unequivocal IAV subtype identification, ideally even in clinical samples, is the prerequisite for monitoring the evolutionary dynamics and diversity of IAVs, with special emphasis on the One Health concept. With the use of RITA, the standard diagnostic cascade recommended for the detection and characterization of IAV in avian samples is reduced to a pretesting using a pan-influenza IAV-assay to verify presence of IAV RNA in the sample and a single RITA run. Future versions of RITA are intended to hold an “avian RITA”, which includes cleavage site-specific assays for H5/H7 pathotyping and the detection of avian pathogens of differential diagnostic relevance. An envisaged “mammalian Rita” can include assays for the differential diagnosis of influenza-like illnesses, while certain “avian” subtypes can be excluded from the array.

## Additional Information

**How to cite this article**: Hoffmann, B. *et al.* Riems influenza a typing array (RITA): An RT-qPCR based low density array for subtyping avian and mammalian influenza a viruses. *Sci. Rep.* **6**, 27211; doi: 10.1038/srep27211 (2016).

## Supplementary Material

Supplementary Information

## Figures and Tables

**Table 1:** Condensed overview of the haemagglutinin subtype of viral isolates used to validate RITA.

	human	avian	Porcine	other mammals	Total
H1	21	10	23	–	54
H2	1	17	–	–	18
H3	8	24	7	1	40
H4	–	17	–	–	17
H5	3	83	–	2	88
H6	–	61	–	–	61
H7	–	34	–	1	35
H8	–	3	–	–	3
H9	–	31	–	–	31
H10	–	29	–	–	29
H11	–	11	–	–	11
H12	–	1	–	–	1
H13	–	9	–	1	10
H14	–	1	–	–	1
H15	–	1	–	–	1
H16	–	4	–	–	4
Total	33	336	30	5	404

**Table 2:** Summary of the neuraminidase subtypes of the viral isolates used to validate RITA.

	N1	N2	N3	N4	N5	N6	N7	N8	N9	total
H1	47	7	–	–	–	–	–	–	–	54
H2	–	2	10	–	–	–	–	–	6	18
H3	1	22	–	–	–	3	–	14	–	40
H4	–	1	–	–	–	16	–	–	–	17
H5	60	9	15	–	–	1	–	–	3	88
H6	7	41	–	1	3	–	–	8	1	61
H7	8	2	7	2	–	–	16	–	–	35
H8	–	–	1	2	–	–	–	–	–	3
H9	–	31	–	–	–	–	–	–	–	31
H10	–	1	–	6	–	–	21	1	–	29
H11	3	1	–	–	–	2	–	–	5	11
H12	–	–	–	–	1	-	–	–	–	1
H13	–	4	–	–	–	4	–	2	–	10
H14	–	–	–	–	1	–	–	–	–	1
H15	–	–	–	–	–	–	–	–	1	1
H16	–	–	4	–	–	–	–	–	–	4
Total	126	121	37	11	5	26	37	25	16	404

**Table 3:** Primers and probes used in this study.

Designation	Sequence 5′⇒ 3′	Concentration of primer and probes in the primer-probe-mix
*Pan-IAV assay*		
IAV-M1.2	Hoffmann *et al.*^15^	
IAV-M1-F	AGA TGA GTC TTC TAA CCG AGG TCG	20 μM
IAV-M1.1-R	TGC AAA AAC ATC TTC AAG TYT CTG	15 μM
IAV-M1.2-R	TGC AAA GAC ACT TTC CAG TCT CTG	15 μM
IAV-M1-FAM	FAM-TCA GGC CCC CTC AAA GCC GA-BHQ1	2.5 μM
*H1 assays*		
IAV-H1-Mix 3-FAM	(this study)	
IAV-H1-115F	ACA CAA TAT GTA TAG GYT AHC ATG C	20 μM
IAV-H1-199R	GAG TGT GTY ACT GTY ACA TTC TT	20 μM
IAV-H1-147FAM	FAM-TCD ACM GAC ACT GTW GAC ACA GTA CTN GA-BHQ1	5 μM
IAV-H1-Mix 27-FAM	(this study)	
IAV-H1-1078F	AGG AAT GTC CCR TCY ATT CAA TC	20 μM
IAV-H1-1086F	CCC GTC YAT TCA ATC YAG AGG	20 μM
IAV-H1-1180R	GGT GAT AAC CRT ACC ANC CAT C	20 μM
IAV-H1-1190R	TCA TTT TGA TGR TGA TAA CCR TAC CA	20 μM
IAV-H1-1155.1FAM	FAM-CAT YCC WGT CCA YCC YCC TTC AAT GAA-BHQ1	5 μM
*H2 assays*		
IAV-H2-Mix 4-FAM	(this study)	
IAV-H2-470-F	GAC ACA GCA YAC RAC AAC TGG	15 μM
IAV-H2-601-R	GTG TTG TTG TAT GAT CYT TTR GCA A	15 μM
IAV-H2-522-FAM	FAM-CCN TCA TTC TTC AGG AAC ATG GTY TGG-BHQ1	5 μM
IAV-H2-Mix 10-FAM	(this study)	
IAV-H2-1075-F	CAA GRG GAT TGT TTG GRG CAA T	15 μM
IAV-H2-1175-R	TGA TCC YTG RTC ATT GCT GTG	15 μM
IAV-H2-1142-FAM	FAM-CCA RCC ATC AAC CAT YCC TTG CCA TCC-BHQ1	5 μM
*H3 assays*		
IAV-H3-Mix 3-FAM	(this study)	
IAV-H3-876-F	CTC CTC GGG GTT AYT TYA AAA T	15 μM
IAV-H3-975-R	CCA TTT GGA GTG ATR CAT TCA GA	15 μM
IAV-H3-934-FAM	FAM-TGC ATC TGA YCT CAT TAT TGA GCT TTT CCC-BHQ1	5 μM
IAV-H3-Mix 14-FAM	(this study)	
IAV-H3-1667-F	TGG ATT TCC TTT GCC ATA TCA TG	15 μM
IAV-H3-1784-R	ATR CAC TCA AAT GCA AAT GTT GCA	15 μM
IAV-H3-1753-FAM	FAM-CTA ATG TTG CCT YTY TGG CAG GCC CAC AT-BHQ1	5 μM
*H4 assay*		
IAV-H4-Mix 15-FAM	(this study)	
IAV-H4-1586F	GAC TCA RGG ATA CAA RGA CAT	15 μM
IAV-H4-1599F	AAG GAC ATC ATY CTY TGG ATT TC	15 μM
IAV-H4-1686R	ACA AGC CCA CAA AAT RAA GGC	15 μM
IAV-H4-1696R	TTC CRT TYT GAC AAG CCC ACA A	15 μM
IAV-H4-1628FAM	FAM-TCC ATA TCA TGC TTY TTR CTC GTT GC-BHQ1	5 μM
*H5 assays*		
IAV-H5-Mix 1-FAM	modified Spackman *et al.*^25^ CRL Weybridge	
IAV-H5-1F	ACA TAT GAC TAC CCA CAR TAT TCA G	25 μM
IAV-H5-1R	AGA CCA GCT AYC ATG ATT GC	25 μM
IAV-H5-1FAM	Fam-TCW ACA GTG GCG AGT TCC CTA GCA-BHQ1	5 μM
IAV-H5a-Mix 1-FAM	(this study)	
IAV-H5a-1658F	GTT CCC TAG YAC TGG CAA TCA T	20 μM
IAV-H5a-1747R	AAT TCT ARA TGC AAA TTC TGC AYT G	20 μM
IAV-H5a-1685FAM	FAM-CTG GTC TAT CYT THT GGA TGT GYT CCA ATG-BHQ1	5 μM
*H6 assay*		
IAV-H6-Mix 8-FAM-MGB	(this study)	
IAV-H6-1666F	CTT GGT GTG TAT CAA ATY CTT GC	20 μM
IAV-H6-1776R	CAT TGA RCC ATT TGA RCA CAT CCA	20 μM
IAV-H6-1693FAM-MGB	FAM-TAT AGT ACG GTA TCG AGC AGY CT-MGB	5 μM
*H7 assays*		
FLI-H7generic-2	Kalthoff *et al.*^23^	
IAV-HA7-1593-F	AYA GAA TAC AGA TWG ACC CAG T	20 μM
IAV-HA7-1740-R	TAG TGC ACY GCA TGT TTC CA	20 μM
IAV-HA7-1649-FAM	FAM-TGG TTT AGC TTC GGG GCA TCA TG –BHQ1	2.5 μM
IAV-H7-2.2-Mix-FAM	(this study)	
IAV-HA7-1617-F	AAA TTG AGC AGY GGM TAC AAR GA	25 μM
IAV-HA7-1707-R	AAA ACC ART CCC ATT RCA ATG GC	25 μM
IAV-HA7-1649.1-FAM	FAM-TGG TTT AGC TTC GGG GCR TCA TGY TT_BHQ1	5 μM
FLI-H7-CODA	van Borm *et al.*^26^	
IAV-HA7-CODA-F	GYA GYG GYT ACA AAG ATG TG	20 μM
IAV-HA7-CODA-R	GAA GAC AAG GCC CAT TGC AA	20 μM
IAV-HA7-CODA-FAM	FAM-TGG TTT AGC TTC GGG GCA TCA TG-BHQ1	2.5 μM
*H8 assay*		
IAV-H8-Mix 1-FAM	(this study)	
IAV-H8-1604F	TAC AAA ATT CTY AGC ATY TAC AGT AC	20 μM
IAV-H8-1677R	ATT ARA CCT CCA GCA AYC AGG A	20 μM
IAV-H8-1654FAM	FAM-TGC CAA GCA RAG ACT GGC CGC CA-BHQ1	5 μM
*H9 assay*		
IAV-H9-Mix 2-FAM	modified Monne *et al.*^24^	
IAV-H9-2F	ATG GGG TTT GCT GCC	20 μM
IAV-H9-2R	TTA TAT ACA AAT GTT GCA YCTG	20 μM
IAV-H9-2FAM	FAM-TTC TGG GCC ATG TCC AAT GG-BHQ1	5 μM
*H10 assays*		
IAV-H10-Mix 5-FAM	(this study)	
IAV-H10-991F	GTT GCT TGC WAC MGG AAT GAG	15 μM
IAV-H10-1041F	GCC TGT TTG GDG CRA TAG C	15 μM
IAV-H10-1122R	TTT TGR TGT CKG AAR CCA TAC CA	15 μM
IAV-H10-1173R	ATA GCT GCY TGA GTA CTY TTG TA	15 μM
IAV-H10-1092FAM	FAM-ACC ATY CCT TCC CAT CCR TTY TCT A-BHQ1	6.25 μM
IAV-H10-Mix 9-FAM	(this study)	
IAV-H10-1567F	GAA ACT CTC TTC TGG VTA YAA AGA	20 μM
IAV-H10-1695R	ATT GTG CAT CGC ATG TTT CCA T	20 μM
IAV-H10-1601FAM	FAM-TGG TTT AGC TTC GGG GCR TCA TGY TT-BHQ1	5 μM
*H11 assay*		
IAV-H11-Mix 1-FAM	(this study)	
IAV-H11-1510F	ARG TYA GGA ATG GAA CAT ATG AYC A	20 μM
IAV-H11-1723R	CAA ATG GTA CAT CTA CAT GAY CCA	20 μM
IAV-H11-1626FAM	FAM-ATT TAC AGC TGC ATY GCA AGY AGT CT_BHQ1	12.5 μM
*H12 assay*		
IAV-H12-Mix 3-FAM	(this study)	
IAV-H12-1607F	AGC ATC TAC AGC AGT GTY GC	20 μM
IAV-H12-1707R	CAG AAA GTA CAA CGA AYA TTT CCA	20 μM
IAV-H12-1674FAM	FAM-CCG AAA ATG AAA CCC CCA ATA ATC ATG A-BHQ1	5 μM
*H13 assay*		
IAV-H13-Mix 2-FAM	(this study)	
IAV-H13-1241F	ATT GAC AAA ATG AAT GGR AAY TAT GAY TC	20 μM
IAV-H13-1372R	AAG AAG YTT DGC ATT RTA TGA CCA	20 μM
IAV-H13-1304FAM	FAM-ATA AAY ATG CTY GCA GAY AGR ATA GAT GAY GC-BHQ1	5 μM
*H16 assay*		
IAV-H16-Mix 6-FAM-MGB	(this study)	
IAV-H16-1589F	GGG ATA AAR TTG AAR ACT GAR GA	20 μM
IAV-H16-1708R	ACT GCT RCA TGC CCA CAK TAT	20 μM
IAV-H16-1635FAM-MGB	FAM-TTT AYA GYT GCA TTG CAA GCA G-MGB	5 μM
*N1 assay*		
IAV-N1-Mix 3-FAM	(this study)	
IAV-N1-3-F	AGR CCT TGY TTC TGG GTT GA	25 μM
IAV-N1-3-R	ACC GTC TGG CCA AGA CCA	25 μM
IAV-N1-3-FAM	FAM-ATY TGG ACY AGT GGG AGC AGC AT-BHQ1	5 μM
*N2 assay*		
IAV-N2-Mix 5.5 FAM-MGB	(this study)	
IAV-N2-1367F	AGT CTG GTG GAC YTC AAA YAG	30 μM
IAV-N2-1488R	AAT TGC GAA AGC TTA TAT AGV CAT	30 μM
IAV-N2-1444.1FAM-MGB	FAM-CCA TCA GGC CAT GAG CCT-MGB	5 μM
*N3 assay*		
IAV-N3-Mix 2-FAM	(this study)	
IAV-N3-1348F	AAY AGT ATA GTT ACT TTC TGY GG	20 μM
IAV-N3-1422R	CCA ATG TTR GAA CCA TCH GG	20 μM
IAV-N3-1373FAM	FAM-TAR ACA ATG AAC CTG GAT CGG GVA A-BHQ1	5 μM
IAV-N3-Mix 4-FAM	(this study)	
IAV-N3-1348F	AAY AGT ATA GTT ACT TTC TGY GG	20 μM
IAV-N3-1422R	CCA ATG TTR GAA CCA TCH GG	20 μM
IAV-N3-1440R	TDT TAC TTG GGC ATD AAC CCA AT	20 μM
IAV-N3-1373FAM	FAM-TAR ACA ATG AAC CTG GAT CGG GVA A-BHQ1	5 μM
*N4 assay*		
IAV-N4-Mix 6-FAM	(this study)	
IAV-N4-1335F	GAC YAG TGG TAG TAG YAT YGC	20 μM
IAV-N4-1345F	AGT AGY ATT GCR TTY TGT GGT GTT	20 μM
IAV-N4-1437R	AAA TYA CTT GTC TAT GTC AAA DGG	20 μM
IAV-N4-1387FAM	FAM-TGG TCR TGG CCY GAT GGC GCT CT-BHQ1	5 μM
*N5 assay*		
IAV-N5-Mix 5-FAM	(this study)	
IAV-N5-1322F	AAG AGA GRA CWA GCA TTT GGA C	15 μM
IAV-N5-1353F	CTC CAC KGT RTT TTG TGG TGT	15 μM
IAV-N5-1421R	GGA AGA ATT GCK CCA TCA YC	15 μM
IAV-N5-1426R	CAA AKG GAA GAA TTG CKC CAT CA	15 μM
IAV-N5-1375FAM	FAM-TCM AGT GAG GTC CCA GGR TGG TC-BHQ1	5 μM
*N6 assay*		
IAV-N6-Mix 3-FAM	(this study)	
IAV-N6-10F	AGG GTG AAR ATG AAT CCA AAY CA	20 μM
IAV-N6-14F	TGA ARA TGA ATC CAA ATC AGA AGA TAA	20 μM
IAV-N6-97R	CAA TCC TAT YAG CAG RCT TAC TAC	20 μM
IAV-N6-43FAM	FAM-TGC ATH TCA GCH ACA GGA ATG ACA CTA TC-BHQ1	5 μM
*N7 assay*		
IAV-N7-Mix 1-FAM	(this study)	
IAV-N7-1305F	GTT GAA TTA ATW AGA GGA AGR CC	20 μM
IAV-N7-1430R	GAT YTG TGC CCC ATC RGG GA	20 μM
IAV-N7-1383FAM	FAM-AGC CCA DTC YCA GTT GGG TCY GGT TC-BHQ1	5 μM
*N8 assay*		
IAV-N8-Mix 1-FAM	(this study)	
IAV-N8-1296F	TCC ATG YTT TTG GGT TGA RAT GAT	20 μM
IAV-N8-1423R	GCT CCA TCR TGC CAY GAC CA	20 μM
IAV-N8-1354FAM	FAM-TCH AGY AGC TCC ATT GTR ATG TGT GGA GT-BHQ1	10 μM
*N9 assay*		
IAV-N9-Mix 11-FAM	Kalthoff *et al.*^23^	
IAV-N9-1363F	AGY ATA GTA TCR ATG TGT TCC AG	20 μM
IAV-N9-1439R	AAG TAC TCT ATT TTA GCC CCA TC	20 μM
IAV-N9-1393FAM	FAM-TTC CTB GGA CAA TGG AAC TGG CC-BHQ1	5 μM
*IC assay*		
EGFP-Mix2- HEX	Hoffmann *et al.*^16^	
EGFP-1-F	GAC CAC TAC CAG CAG AAC AC	5 μM
EGFP-10-R	CTT GTA CAG CTC GTC CAT GC	5 μM
EGFP-HEX	HEX-AGC ACC CAG TCC GCC CTG AGC A-BHQ1	3.75 μM

**Table 4:** Reactivity of HA subtype-specific RT-qPCR assays used in RITA with the validation panel of 404 viral isolates.

RT-qPCR	H1	H2	H3	H4	H5	H6	H7	H8	H9	H10	H11	H12	H13	H14	H15	H16
IAV-H1-Mix 3	51	–	–	–	–	–	–	–	–	–	–	–	-	–	–	–
IAV–H1–Mix 27	53	–	–	–	–	37*	–	3*	–	–	–	–	–	–	–	–
IAV–H2–Mix 4	–	18	–	–	–	–	–	–	–	–	–	–	–	–	–	–
IAV–H2–Mix 10	–	18	–	–	1*	-	–	–	–	–	–	–	–	–	-	–
IAV–H3–Mix 3	–	–	35	–	–	–	–	–	–	–	–	–	–	–	–	–
IAV–H3–Mix 14	–	–	40	–	–	–	–	–	–	–	–	–	–	–	–	–
IAV–H4–Mix 15	–	–	–	17	–	–	–	–	–	–	–	–	–	–	–	–
IAV-H5-Mix 1	–	–	–	–	87	–	–	–	–	–	–	–	–	–	–	–
IAV–H5a–Mix 1	–	–	–	–	88	–	–	–	–	–	–	–	–	–	–	–
IAV-H6-Mix 8	–	–	–	–	–	61	–	–	–	–	–	–	–	–	–	–
IAV-H7-CODA	–	–	–	–	1*	–	33	–	–	1*	–	–	–	–	1*	–
IAV–H7-Mix 2	–	–	–	–	–	–	35	–	–	6*	–	–	––	–	–	–
IAV-H7-Mix 2.2	–	–	–	–	–	–	35	–	–	–	–	–	–	–	–	–
IAV-H8-Mix 1	–	–	–	–	–	–	–	3	–	–	–	–	–	–	–	–
IAV-H9-Mix 2	–	–	–	–	–	–	–	–	31	–	–	–	–	–	–	–
IAV-H10-Mix 5	–	–	30#	–	–	–	–	–	–	29	–	–	–	–	–	–
IAV-H10-Mix 9	–	–	–	–	–	–	30†	–	–	29	–	–	–	–	–	–
IAV-H11-Mix 1	–	–	–	–	–	–	–	–	–	–	11	–	–	–	–	–
IAV-H12-Mix 3	–	–	–	–	–	–	–	–	–	–	–	1	–	–	–	–
IAV-H13-Mix 2	–	–	–	–	–	–	–	–	–	–	–	–	10	–	–	–
IAV-H16-Mix 6	–	–	–	–	–	–	–	–	–	–	–	–	–	–	–	4
total	54	18	40	17	88	61	35	3	31	29	11	1	10	1	1	4

^*^Δ C_q_ between pan assay and false positive reactivity ≥10 and Δ C_q_ between specific assay and false positive reactivity ≥8.

^#^Δ C_q_ between pan assay and false positive reactivity ≥3 and Δ C_q_ between specific assay and false positive reactivity ≥3.

^†^Δ C_q_ between pan assay and false positive reactivity ≥5; and Δ C_q_ between specific assay and false positive reactivity ≥4.

**Table 5:** Reactivity of NA subtype-specific RT-qPCR assays used in RITA with the validation panel of 404 viral isolates.

RT-qPCR	N1	N2	N3	N4	N5	N6	N7	N8	N9
IAV-N1-Mix 3	122	–	–	–	–	–	–	–	–
IAV-N2-Mix 5.5	–	118	–	–	–	–	–	–	–
IAV-N3-Mix 2	–	–	37	–	–	–	–	–	–
IAV-N3-Mix 4	–	–	37	–	–	–	–	–	–
IAV-N4-Mix 6	–	–	–	11	–	–	–	–	–
IAV-N5-Mix 5	–	–	–	–	6	–	–	–	–
IAV-N6-Mix 3	–	–	–	–	–	26	–	–	–
IAV-N7-Mix 1	–	–	–	–	–	–	37	–	–
IAV-N8-Mix 1	–	–	–	–	–	–	–	25	–
IAV-N9-Mix 11	–	–	–	–	–	–	–	–	16
total	122	118	37	11	6	26	37	25	16

**Table 6:** Diagnostic validation of RITA results from IAV-positive avian and porcine swab samples.

Sample origin	total no. tested	Subtyping by RITA							Discrepancy RITA subtyping and pan-HA/NA sequencing+
		specific results		presumed non-specific reactivity#		mixed infection	Subtyping by pan-HA/NA sequencing		
		HA	NA	HA	NA				HA	NA	
wild bird swabs*	45	45	44	16 (11†/5‡)	1 (0/1)	4	32	40	0
swine nasal swabs	17	17	17	1 (1†/0‡)	0	1	17	16	0
total	62	62	61	17 (12/5)	1 (0/1)	5	49	56	0

^*^oropharyngeal, cloacal or combined swab samples.

^#^RITA-nonspecific results are characterized by Δ Cq values according to details mentioned in table 4.

^†^Nonspecific reactivity also detected with viruses of the validation panel.

^‡^Nonspecific reactivity not previously seen in the validation panel.

^+^In 13 and 6 samples, respectively, RITA could identify additional HA and NA subtypes (one or several) in comparison to the sequencing results. In one sample only the pan-NA sequencing procedure delivered a subtyping result. For more details see table S2.

## References

[b1] Vahlenkamp T. W. & Harder T. C. Influenza virus infections in mammals. Berliner und Munchener tierarztliche Wochenschrift 119, 123–131 (2006). 16573202

[b2] Fields B. N., Knipe D. M. D. M.- & Howley P. M. Fields virology. 6th ed. edn, Vol. 1 (Wolters Kluwer, Lippincott Williams & Wilkins, 2013).

[b3] Urbaniak K. & Markowska-Daniel I. *In vivo* reassortment of influenza viruses. Acta Biochim Pol 61, 427–431 (2014). 25180223

[b4] A revision of the system of nomenclature for influenza viruses: a WHO memorandum. Bulletin of the World Health Organization 58, 585–591 (1980). 6969132 PMC2395936

[b5] Fouchier R. A. *et al.* Characterization of a novel influenza A virus hemagglutinin subtype (H16) obtained from black-headed gulls. Journal of virology 79, 2814–2822, 10.1128/JVI.79.5.2814-2822.2005 (2005). 15709000 PMC548452

[b6] Webster R. G. & Bean W. J. Jr. Genetics of influenza virus. Annual review of genetics 12, 415–431, 10.1146/annurev.ge.12.120178.002215 (1978). 371528

[b7] Tong S. *et al.* A distinct lineage of influenza A virus from bats. Proceedings of the National Academy of Sciences of the United States of America 109, 4269–4274, 10.1073/pnas.1116200109 (2012). 22371588 PMC3306675

[b8] Tong S. *et al.* New world bats harbor diverse influenza A viruses. PLoS Pathog 9, e1003657, 10.1371/journal.ppat.1003657 (2013). 24130481 PMC3794996

[b9] Influenza (seasonal) Fact sheet N°211, http://who.int/mediacentre/factsheets/fs211/en, (Date of access: 27/04/2016) (2014).

[b10] Girard M. P., Cherian T., Pervikov Y. & Kieny M. P. A review of vaccine research and development: human acute respiratory infections. Vaccine 23, 5708–5724, 10.1016/j.vaccine.2005.07.046 (2005). 16154667 PMC7130922

[b11] OIE. Manual of Diagnostic Tests and Vaccines for Terrestrial Animals, http://www.oie.int/fileadmin/Home/fr/Health_standards/tahm/2.03.04_AI.pdf, (Date of access: 27/04/2016) (2015).

[b12] Nagy A. *et al.* MeltMan: Optimization, Evaluation, and Universal Application of a qPCR System Integrating the TaqMan qPCR and Melting Analysis into a Single Assay. PloS one 11, e0151204, 10.1371/journal.pone.0151204 (2016). 27031831 PMC4816343

[b13] Gall A. *et al.* Rapid and highly sensitive neuraminidase subtyping of avian influenza viruses by use of a diagnostic DNA microarray. Journal of clinical microbiology 47, 2985–2988, 10.1128/JCM.00850-09 (2009). 19587298 PMC2738067

[b14] Gall A. *et al.* Rapid haemagglutinin subtyping and pathotyping of avian influenza viruses by a DNA microarray. Journal of virological methods 160, 200–205, 10.1016/j.jviromet.2009.05.004 (2009). 19447139

[b15] Hoffmann B. *et al.* New real-time reverse transcriptase polymerase chain reactions facilitate detection and differentiation of novel A/H1N1 influenza virus in porcine and human samples. Berliner und Munchener tierarztliche Wochenschrift 123, 286–292 (2010). 20690540 10.2376/0005-9366-123-286

[b16] Hoffmann B., Depner K., Schirrmeier H. & Beer M. A universal heterologous internal control system for duplex real-time RT-PCR assays used in a detection system for pestiviruses. Journal of virological methods 136, 200–209, 10.1016/j.jviromet.2006.05.020 (2006). 16806503

[b17] Hoffmann B. *et al.* Rapid and highly sensitive pathotyping of avian influenza A H5N1 virus by using real-time reverse transcription-PCR. Journal of clinical microbiology 45, 600–603, 10.1128/JCM.01681-06 (2007). 17182758 PMC1829000

[b18] Briand F. X., Niqueux E., Brochet A. L., Hars J. & Jestin V. Unusual H5N2 avian influenza virus escapes current detection. Journal of clinical microbiology 49, 2376–2377, 10.1128/JCM.00479-11 (2011). 21471334 PMC3122761

[b19] Elizalde M. *et al.* Rapid molecular haemagglutinin subtyping of avian influenza isolates by specific real-time RT-PCR tests. Journal of virological methods 196, 71–81, 10.1016/j.jviromet.2013.10.031 (2014). 24184949

[b20] Johnson M. P., Haupt L. M. & Griffiths L. R. Locked nucleic acid (LNA) single nucleotide polymorphism (SNP) genotype analysis and validation using real-time PCR. Nucleic Acids Res 32, e55, 10.1093/nar/gnh046 (2004). 15047860 PMC390376

[b21] Tsukamoto K. *et al.* SYBR green-based real-time reverse transcription-PCR for typing and subtyping of all hemagglutinin and neuraminidase genes of avian influenza viruses and comparison to standard serological subtyping tests. Journal of clinical microbiology 50, 37–45, 10.1128/JCM.01195-11 (2012). 22031706 PMC3256701

[b22] Latorre-Margalef N. *et al.* Long-term variation in influenza A virus prevalence and subtype diversity in migratory mallards in northern Europe. Proc Biol Sci 281, 20140098, 10.1098/rspb.2014.0098 (2014). 24573857 PMC3953854

[b23] Kalthoff D. *et al.* Nucleic acid-based detection of influenza A virus subtypes H7 and N9 with a special emphasis on the avian H7N9 virus. Euro surveillance : bulletin Europeen sur les maladies transmissibles = European communicable disease bulletin 19 **(10)**, pii = 20731, 10.2807/1560-7917.ES2014.19.10.20731 (2014). 24650867

[b24] Monne I. *et al.* Development and validation of a one-step real-time PCR assay for simultaneous detection of subtype H5, H7, and H9 avian influenza viruses. Journal of clinical microbiology 46, 1769–1773, 10.1128/JCM.02204-07 (2008). 18367569 PMC2395090

[b25] Spackman E. *et al.* Development of a real-time reverse transcriptase PCR assay for type A influenza virus and the avian H5 and H7 hemagglutinin subtypes. Journal of clinical microbiology 40, 3256–3260 (2002). 12202562 10.1128/JCM.40.9.3256-3260.2002 PMC130722

[b26] Van Borm S. *et al.* Rapid detection of Eurasian and American H7 subtype influenza A viruses using a single TaqManMGB real-time RT-PCR. Avian diseases 54, 632–638 (2010). 20521706 10.1637/8734-032509-ResNote.1

[b27] Das A. & Suarez D. L. Development and bench validation of real-time reverse transcription polymerase chain reaction protocols for rapid detection of the subtypes H6, H9, and H11 of avian influenza viruses in experimental samples. Journal of veterinary diagnostic investigation : official publication of the American Association of Veterinary Laboratory Diagnosticians, Inc 19, 625–634 (2007). 10.1177/104063870701900603 17998550

[b28] Dugan V. G. *et al.* The evolutionary genetics and emergence of avian influenza viruses in wild birds. PLoS Pathog 4, e1000076, 10.1371/journal.ppat.1000076 (2008). 18516303 PMC2387073

[b29] Rejmanek D., Hosseini P. R., Mazet J. A., Daszak P. & Goldstein T. Evolutionary Dynamics and Global Diversity of Influenza A Virus. Journal of virology 89, 10993–11001, 10.1128/JVI.01573-15 (2015). 26311890 PMC4621101

