# Supplementary material for: Riems influenza a typing array (RITA): An RT-qPCR-based low density array for subtyping avian and mammalian influenza a viruses
Source: Sci Rep. 2016 Jun 3;6:27211. doi: 10.1038/srep27211 (PMC4891686; doi:10.1038/srep27211)

RIEMS INFLUENZA A TYPING ARRAY (RITA): AN RT-QPCR BASED LOW DENSITY ARRAY FOR SUBTYPING AVIAN AND MAMMALIAN INFLUENZA A VIRUSES

Bernd Hoffmann, Donata Hoffmann*, Dinah Henritzi, Martin Beer and Timm C. Harder

Supplementary table 1: Details of individual strains included into the validation panel of RITA.

| **Isolate** | **sub-typing** | | **HA, NA sequence accession** | **Host species** |
| --- | --- | --- | --- | --- |
|  | **HA-type** | **NA-type** |  |  |
|  |  |  |  |  |
| A/Puerto Rico/8/1934 (H1N1) | **H1** | **N1** | EF467821, EF467823 | human |
| A/FM/1/47 (H1N1) | **H1** | **N1** | U02464, AF250357 | human |
| A/England/1/51 (H1N1) | **H1** | **N1** | not available | human |
| A/Denver/1957 (H1N1) | **H1** | **N1** | CY008988, CY008990 | human |
| A/USSR/90/77 (H1N1) | **H1** | **N1** | CY010372, CY010374 | human |
| A/Chile/1/83 (H1N1) | **H1** | **N1** | X15281 | human |
| A/Taiwan/1/1986 (H1N1) | **H1** | **N1** | D00407, KF356051 | human |
| A/New Caledonia/20/1999 (H1N1) | **H1** | **N1** | CY125100, CY125102 | human |
| A/Niedersachsen/14/2007 (H1N1) | **H1** | **N1** | not available | human |
| A/Baden-Wuerttemberg/200/2007 (H1N1) | **H1** | **N1** | not available | human |
| A/Rheinland-Pfalz/14/2007 (H1N1) | **H1** | **N1** | not available | human |
| A/ Nordrhein-Westfalen/1/2007 (H1N1) | **H1** | **N1** | EPI509298, EPI509299 | human |
| A/Saarland/2/2007 (H1N1) | **H1** | **N1** | not available | human |
| A/Thüringen/3/2007 (H1N1) | **H1** | **N1** | not available | human |
| A/California/7/2009 (H1N1pdm) | **H1pdm** | **N1** | NC026433, NC026434 | human |
| A/Regensburg/D6/2009 (H1N1pdm) | **H1pdm** | **N1** | FN401574, FN401575 | human |
| A/Germany-BY/74/2009 (H1N1pdm**)** | **H1pdm** | **N1** | HM138501, HM138500 | human |
| A/Hamburg/4/2009 (H1N1pdm) | **H1pdm** | **N1** | GQ166213, GQ166217 | human |
| A/Germany/HGW1/2009 (H1N1pdm) | **H1pdm** | **N1** | not available | human |
| A/Germany-MV/HGW4/2009 (H1N1pdm) | **H1pdm** | **N1** | HM138491, HM138490 | human |
| A/Germany-MV/HGW6/09 (H1N1pdm) | **H1pdm** | **N1** | HM138493, HM138492 | human |
| A/duck/Bavaria/1/77 (H1N1) | **H1** | **N1** | AF091313 | avian |
| A/duck/Germany/R30/2006 (H1N1) | **H1** | **N1** | not available | avian |
| A/Anser egypticus/Germany/R1419/2006 (H1N1) | **H1** | **N1** | AM922139 | avian |
| A/mallard/Germany/R3036/2007 (H1N1) | **H1** | **N1** | not available | avian |
| A/wild duck/Germany-NW/R04/2008 (H1N1) | **H1** | **N1** | EPI248521, EPI248520 | avian |
| A/mallard/Germany/R292/2008 (H1N1) | **H1** | **N1** | not available | avian |
| A/mallard/Germany-RP/R193/2009 (H1N1) | **H1** | **N1** | EPI248501, EPI248500 | avian |
| A/white fronted geese/Germany/R481/2009 (H1N1) | **H1** | **N1** | not available | avian |
| A/white-front goose/Germany-NI/R482/2009 (H1N1) | **H1** | **N1** | EPI248525, EPI248524 | avian |
| A/turkey/Ontario/FAV110-4/2009 (H1N1pdm) | **H1pdm** | **N1** | HM370967, HM370969 | avian |
| A/swine/Potsdam/1/1981 (H1N1) | **H1** | **N1** | KJ889379, KJ889381 | porcine |
| A/swine/Belzig/2/2001 (H1N1) | **H1** | **N1** | EPI302495, EPI302494 | porcine |
| A/swine/Greven/IDT2889/2004 (H1N1) | **H1** | **N1** | GQ161156, GQ161158 | porcine |
| A/swine/Bakum/3543/2008 | **H1** | **N1** | not available | porcine |
| A/swine/Germany-NI/R3394/2009 (H1N1) | **H1** | **N1** | EPI278647, EPI278646 | porcine |
| A/swine/Germany-BB/siv-leipz11308/2009 (H1N1) | **H1** | **N1** | EPI248493, EPI248492 | porcine |
| A/swine/Germany-NI/R211/2009 (H1N1) | **H1** | **N1** | EPI278638, EPI278637 | porcine |
| A/swine/Germany-SN/siv-leipz6340/2009 (H1N1) | **H1** | **N1** | EPI248505, EPI248504 | porcine |
| A/swine/Germany-TH/R2241/2009 (H1N1) | **H1** | **N1** | EPI278645, EPI278644 | porcine |
| A/wild boar/Germany-HE/ws336/2009 (H1N1) | **H1** | **N1** | EPI396882, EPI396881 | porcine |
| A/swine/Germany/R248/1/2010 (H1N1) | **H1** | **N1** | not available | porcine |
| A/swine/Germany-NW/R708/2010 (H1N1) | **H1** | **N1** | EPI301665, EPI301664 | porcine |
| A/swine/Germany/AR1154/2014 | **H1** | **N1** | not available | porcine |
| A/swine/France/AR1348/2015 | **H1** | **N1** | not available | porcine |
| A/swine/Denmark/AR1855/2015 | **H1pdm** | **N1** | not available | porcine |
| A/swine/Netherlands/AR2056/2015 | **H1** | **N1** | not available | porcine |
| A/swine/Scotland/410440/1994 (H1N2) | **H1** | **N2** | AF085413, AJ412697 | porcine |
| A/swine/Bakum/1832/2000 (H1N2) | **H1** | **N2** | GQ161104, GQ161106 | porcine |
| A/swine/Granstedt/IDT3475/2004 (H1N2) | **H1** | **N2** | GQ161163, GQ161165 | porcine |
| A/swine/Germany-NI/R299/2009 (H1N2) | **H1** | **N2** | EPI278734, EPI278733 | porcine |
| A/swine/Germany-NI/R369/2009 (H1N2) | **H1** | **N2** | EPI278736, EPI278735 | porcine |
| A/swine/Germany/AR1703/2014 | **H1** | **N2** | not available | porcine |
| A/swine/Spain/AR1372/2015 | **H1** | **N2** | not available | porcine |
| A/Singapore/1/1957 (H2N2) | **H2** | **N2** | CY125894, CY125896 | human |
| A/bantam/Germany/DZ4/85 (H2N2) | **H2** | **N2** | AM922141 | avian |
| A/mallard/Germany/Wv1226-30/2003 (H2N3) | **H2** | **N3** | not available | avian |
| A/mallard/Germany/Wv1231-34/2003 (H2N3) | **H2** | **N3** | not available | avian |
| A/mallard/Germany/Wv1317-21/2003 (H2N3) | **H2** | **N3** | AM922142 | avian |
| A/mallard/Germany/Wv481/2004 (H2N3) | **H2** | **N3** | not available | avian |
| A/mallard/Germany/Wv672/2004 (H2N3) | **H2** | **N3** | not available | avian |
| A/mallard/Germany/Wv677/2004 (H2N3) | **H2** | **N3** | AM922143 | avian |
| A/mallard/Germany/Wv943-45/2004 (H2N3) | **H2** | **N3** | not available | avian |
| A/mallard/Germany/R1169/2006 (H2N3) | **H2** | **N3** | not available | avian |
| A/Mallard/Germany/SR517/2007 (H2N3) | **H2** | **N3** | EPI222785, EPI359509 | avian |
| A/mallard/Germany/R1727/2007 (H2N3) | **H2** | **N3** | not available | avian |
| A/mute swan/Germany/R3227/2007 (H2N3) | **H2** | **N3** | not available | avian |
| A/mallard/Germany/R2711/07 (H2N9) | **H2** | **N9** | not available | avian |
| A/wild duck/Germany/R3111/2007 (H2N9) | **H2** | **N9** | not available | avian |
| A/Greylag goose/Germany/R1416/2008 (H2N9) | **H2** | **N9** | not available | avian |
| A/Greylag goose/Germany/R1485/2008 (H2N9) | **H2** | **N9** | not available | avian |
| A/Greylag goose/Germany/R1486/2008 (H2N9) | **H2** | **N9** | not available | avian |
| A/Greylag goose/Germany/R1487/2008 (H2N9) | **H2** | **N9** | not available | avian |
| A/wild duck/Germany/R2555/2006 (H3N1) | **H3** | **N1** | not available | avian |
| A/HongKong/1/1968 (H3N2) | **H3** | **N2** | AM922145 | Human |
| A/England/42/1972 (H3N2) | **H3** | **N2** | AF201875, AY210132 | Human |
| A/Port Chalmers/1/1973 (H3N2) | **H3** | **N2** | CY009348, CY009350 | Human |
| A/Victoria/3/1975 (H3N2) | **H3** | **N2** | CY113181, CY113183 | Human |
| A/Texas/1/1977 (H3N2) | **H3** | **N2** | CY113261, CY113263 | Human |
| A/Bangkok/1/1979 (H3N2) | **H3** | **N2** | CY121000, CY121002 | Human |
| A/Sichuan/2/1987 (H3N2) | **H3** | **N2** | CY121293, CY121295 | Human |
| A/Johannesburg/33/1994 (H3N2) | **H3** | **N2** | CY121341; CY121343 | Human |
| A/mallard/Germany/Wv436/2004 (H3N2) | **H3** | **N2** | not available | avian |
| A/mallard/Germany/Wv437/2004 (H3N2) | **H3** | **N2** | EPI185346, EPI185340 | avian |
| A/mallard/Germany/Wv438/2004 (H3N2) | **H3** | **N2** | not available | avian |
| A/mallard/Germany/Wv439/2004 (H3N2) | **H3** | **N2** | not available | avian |
| A/mallard/Germany-BW/SR519/2007 (H3N2) | **H3** | **N2** | EPI279919 | avian |
| A/mallard/Germany-BW/SR520/2007 (H3N2) | **H3** | **N2** | EPI279920 | avian |
| A/mallard/Germany-BW/SR632/2008 (H3N2) | **H3** | **N2** | EPI279922 | avian |
| A/swine/Karrenzin/1/1987 (H3N2) | **H3** | **N2** | not available | porcine |
| A/swine/Bakum/909/1993 (H3N2) | **H3** | **N2** | AJ252130, EF409255 | porcine |
| A/swine/Lohne/1/1997 (H3N2) | **H3** | **N2** | EF409249, EF409257 | porcine |
| A/swine/Bakum/1362/1998 (H3N2) | **H3** | **N2** | not available | porcine |
| A/swine/Belzig/54/2001 (H3N2) | **H3** | **N2** | not available | porcine |
| A/swine/Bissendorf/IDT1864/2003 (H3N2) | **H3** | **N2** | GQ161170, GQ161172 | porcine |
| A/swine/Netherlands/AR543/2015 | **H3** | **N2** | not available | porcine |
| A/mallard/Germany/R2619/2007 (H3N6) | **H3** | **N6** | not available | avian |
| A/mallard/Germany/R714/2008 (H3N6) | **H3** | **N6** | not available | avian |
| A/mallard/Germany/R752/2008 (H3N6) | **H3** | **N6** | EPI185338, EPI185344 | avian |
| A/mallard/Germany/Wv1303-04/2003 (H3N8) | **H3** | **N8** | not available | avian |
| A/mallard/Föhr/Wv548/2004 (H3N8) | **H3** | **N8** | not available | avian |
| A/mallard/Germany/Wv50/2005 (H3N8) | **H3** | **N8** | not available | avian |
| A/mallard/Germany/Wv64-67/2005 (H3N8) | **H3** | **N8** | not available | avian |
| A/domestic goose/Germany/R1967/2006 (H3N8) | **H3** | **N8** | not available | avian |
| A/mallard/Germany/R1648/2007 (H3N8) | **H3** | **N8** | not available | avian |
| A/Anas platyrhynchos/Germany/R2322/2007 (H3N8) | **H3** | **N8** | AM930526 | avian |
| A/mallard/Germany/2323/2007 (H3N8) | **H3** | **N8** | not available | avian |
| A/mallard/Germany/R527/2008 (H3N8) | **H3** | **N8** | not available | avian |
| A/mallard/Germany/R529/2008 (H3N8) | **H3** | **N8** | EPI185336, EPI185337 | avian |
| A/mallard/Germany-BE/R663/2008 (H3N8) | **H3** | **N8** | EPI416239, EPI339172 | avian |
| A/mallard/Germany/R682/2008 (H3N8) | **H3** | **N8** | not available | avian |
| A/mallard/Germany/R2379/2009 (H3N8) | **H3** | **N8** | not available | avian |
| A/equine/Miami/1/1963 (H3N8) | **H3** | **N8** | M29257, L06580 | equine |
| A/mallard/Germany/Wv51/2005 (H4N2) | **H4** | **N2** | not available | avian |
| A/mallard/Germany/Wv1754-57/2003 (H4N6) | **H4** | **N6** | AM922148 | avian |
| A/mallard/Germany/Wv1732-34/2003 (H4N6) | **H4** | **N6** | AM922149 | avian |
| A/mallard/Germany/Wv1806-09/03 (H4N6) | **H4** | **N6** | AM922147, AM933235 | avian |
| A/mallard/Germany/Wv1027/2004 (H4N6) | **H4** | **N6** | not available | avian |
| A/mallard/Germany/Wv90/2005 (H4N6) | **H4** | **N6** | not available | avian |
| A/teal/Germany/Wv631/2005 (H4N6) | **H4** | **N6** | not available | avian |
| A/owl/Germany/R31/2006 (H4N6) | **H4** | **N6** | not available | avian |
| A/mallard/Germany/R33/2006 (H4N6) | **H4** | **N6** | not available | avian |
| A/duck/Germany/R2690/2006 (H4N6) | **H4** | **N6** | not available | avian |
| A/swan/Germany/R2925/2006 (H4N6) | **H4** | **N6** | not available | avian |
| A/mallard/Germany/1507/2007 (H4N6) | **H4** | **N6** | not available | avian |
| A/mallard/Germany/1740/1/2007 (H4N6) | **H4** | **N6** | not available | avian |
| A/mallard/Germany/SKo50K/2007 (H4N6) | **H4** | **N6** | not available | avian |
| A/mallard/Germany/R485/3/2008 (H4N6) | **H4** | **N6** | not available | avian |
| A/mallard/Germany/R486/3/2008 (H4N6) | **H4** | **N6** | not available | avian |
| A/mallard/Germany/R726/2008 (H4N6) | **H4** | **N6** | not available | avian |
| A/HongKong/156/1997 (H5N1; HP) | **H5** | **N1** | AF028708, AF028709 | Human |
| A/Hongkong/213/2003 NIBRG12 (H5N1) | **H5** | **N1** | Nicolson, et al. 2005. | Human |
| A/Vietnam/1194/2004 NIBRG14 (H5N1) | **H5** | **N1** | Nicolson, et al. 2005. | Human |
| A/chicken/Indonesien/R132/2003 (H5N1; HP) | **H5** | **N1** | not available | avian |
| A/chicken/Indonesien/R133/2003 (H5N1; HP) | **H5** | **N1** | not available | avian |
| A/chicken/Indonesien/R134/2003 (H5N1; HP) | **H5** | **N1** | AM183669, AM183681 | avian |
| A/chicken/GXLA/12/2004 (H5N1; HP) | **H5** | **N1** | not available | avian |
| A/teal/Germany/Wv632/2005 (H5N1; LP) | **H5** | **N1** | CY061885, CY061887 | avian |
| A/chicken/Indonesien/R60/2005 (H5N1; HP) | **H5** | **N1** | not available | avian |
| A/duck/Vietnam/AG40-O2/2005 (H5N1; HP) | **H5** | **N1** | AM183676 | avian |
| A/chicken/Vietnam/P22/2005 (H5N1; HP) | **H5** | **N1** | AM183674, AM183679 | avian |
| A/chicken/Vietnam/P41/2005 (H5N1; HP) | **H5** | **N1** | AM183672 | avian |
| A/chicken/Vietnam/P78/2005 (H5N1; HP) | **H5** | **N1** | AM183673 | avian |
| A/duck/Vietnam/TG24-01/2005 (H5N1; HP) | **H5** | **N1** | AM183677, AM183678 | avian |
| A/duck/Vietnam/TG36-H2/2005 (H5N1; HP) | **H5** | **N1** | AM183675 | avian |
| A/swan/Germany/R65/2006 (H5N1; HP) | **H5** | **N1** | DQ464354, DQ464355 | avian |
| A/whooper swan/Germany/R88/2006 (H5N1, HP) | **H5** | **N1** | AM403462, AM403135 | avian |
| A/gull/Germany/R139/2006 (H5N1; HP) | **H5** | **N1** | not available | avian |
| A/tufted duck/Germany/R143/2006 (H5N1; HP) | **H5** | **N1** | not available | avian |
| A/cormorant/Germany/R292/2006 (H5N1; HP) | **H5** | **N1** | not available | avian |
| A/tufted duck/Germany/R409/2006 (H5N1; HP) | **H5** | **N1** | not available | avian |
| A/turkey/Turkey/R11/2006 (H5N1; HP) | **H5** | **N1** | not available | avian |
| A/chicken/Turkey/R12/2006 (H5N1; HP) | **H5** | **N1** | not available | avian |
| A/Mergus albellus/Slovakia/Vh212/2006 (H5N1; HP) | **H5** | **N1** | EU443550, EU443566 | avian |
| A/peregrine falcon/Slovakia/Vh246/2006 (H5N1; HP) | **H5** | **N1** | EU443552, EU443565 | avian |
| A/Cygnus olor/Germany/R1377/2007 (H5N1; HP) | **H5** | **N1** | not available | avian |
| A/domestic goose/Germany/R1400/07 (H5N1; HP) | **H5** | **N1** | AM914004, AM914005 | avian |
| A/Great-crested grebe/Germany/R1406/2007 (H5N1; HP) | **H5** | **N1** | AM914012, AM914013 | avian |
| A/Cygnus olor/Germany/R1452/2007 (H5N1; HP) | **H5** | **N1** | not available | avian |
| A/Cygnus olor/Germany/R1454/2007 (H5N1; HP) | **H5** | **N1** | not available | avian |
| A/little grebe/Germany/R1483/2007 (H5N1; HP) | **H5** | **N1** | not available | avian |
| A/black-necked grebe/Germany/R1493/2007 (H5N1; HP) | **H5** | **N1** | not available | avian |
| A/little grebe/Germany/R1525/2007 (H5N1; HP) | **H5** | **N1** | not available | avian |
| A/black-necked grebe/Germany/R1566/2007 (H5N1; HP) | **H5** | **N1** | not available | avian |
| A/duck/Germany/R1772/2007 (H5N1; HP) | **H5** | **N1** | AM914016, AM914017 | avian |
| A/duck/Germany/R1779/2007 (H5N1; HP) | **H5** | **N1** | AM914021, AM914022 | avian |
| A/duck/Germany/R1790/2007 (H5N1; HP) | **H5** | **N1** | not available | avian |
| A/domestic_duck/Germany/R1959/2007 (H5N1; HP) | **H5** | **N1** | AM914026, AM914027 | avian |
| A/duck/Germany/R1970/2007 (H5N1; HP) | **H5** | **N1** | not available | avian |
| A/duck/Germany/R1981/2007 (H5N1; HP) | **H5** | **N1** | not available | avian |
| A/duck/Germany/R2001/2007 (H5N1; HP) | **H5** | **N1** | not available | avian |
| A/duck/Germany/R2014/2007 (H5N1; HP) | **H5** | **N1** | not available | avian |
| A/domestic duck/Germany/R2048/2007 (H5N1; HP) | **H5** | **N1** | AM914026, AM914027 | avian |
| A/duck (meat)/Germany/R2049/2007 (H5N1; HP) | **H5** | **N1** | not available | avian |
| A/duck (meat)/Germany/R2053/2007 (H5N1; HP) | **H5** | **N1** | not available | avian |
| A/chicken/Germany/3294/2007 (H5N1; HP) | **H5** | **N1** | FM177135, FM177137 | avian |
| A/chicken/Poland/52/2007 (H5N1; HP) | **H5** | **N1** | not available | avian |
| A/chicken/Egypt/0815-NLQP/2008 (H5N1; HP) | **H5** | **N1** | GQ184221, GQ184265 | avian |
| A/chicken/Egypt/0827-NLQP/2008 (H5N1; HP) | **H5** | **N1** | not available | avian |
| A/chicken/Egypt/083-NLQP/2008 (H5N1; HP) | **H5** | **N1** | not available | avian |
| A/chicken/Egypt/0832-NLQP/2008 (H5N1; HP) | **H5** | **N1** | not available | avian |
| A/chicken/Egypt/0833-NLQP/2008 (H5N1; HP) | **H5** | **N1** | not available | avian |
| A/chicken/Egypt/0879-NLQP/2008 (H5N1; HP) | **H5** | **N1** | GQ184238, GQ184279 | avian |
| A/duck/Egypt/0897-NLQP/2008 (H5N1; HP) | **H5** | **N1** | JF746738, HQ908448 | avian |
| A/chicken/Egypt/0918-NLQP/2009 (H5N1; HP) | **H5** | **N1** | not available | avian |
| A/duck/Egypt/G2H3-NLQP/2008 (H5N1; HP) | **H5** | **N1** | not available | avian |
| A/chicken/Egypt/G3H4-NLQP/2008 (H5N1; HP) | **H5** | **N1** | not available | avian |
| A/turkey/Poland/36/2008 (H5N1; HP) | **H5** | **N1** | not available | avian |
| A/cat/Germany/R606/2006 (H5N1, HP) | **H5** | **N1** | DQ643982, DQ643984 | mammalian |
| A/stone_marten/Germany/R747/2006 (H5N1; HP) | **H5** | **N1** | AM492165, AM492166 | mammalian |
| A/turkey/Kfar Vitkin/1971 (H5N2; LP) | **H5** | **N2** | not available | avian |
| A/duck/Potsdam/1402/1986 (H5N2; LP) | **H5** | **N2** | CY014642, CY005778 | avian |
| A/duck/Potsdam/1403/1986 (H5N2; ) | **H5** | **N2** | not available | avian |
| A/duck/Potsdam/1701/1986 (H5N2; LP) | **H5** | **N2** | not available | avian |
| A/chicken/Italy/8/1998 (H5N2; HP) | **H5** | **N2** | EF597267, EF597309 | avian |
| A/Teal/Föhr/Wv1378-79/2003 (H5N2;LP) | **H5** | **N2** | not available | avian |
| A/mallard/Germany/Wv474-77K/2004 (H5N2) | **H5** | **N2** | not available | avian |
| A/mallard/Föhr/Wv476/2004 (H5N2; LP) | **H5** | **N2** | not available | avian |
| A/duck/British Columbia/CN26-6/2005 (H5N2; LP) | **H5** | **N2** | DQ309439, DQ309440 | avian |
| A/tern/South Africa/1961 (H5N3; HP) | **H5** | **N3** | CY107861, CY014986 | avian |
| A/mallard/Germany/WV1349/2003 (H5N3, LP) | **H5** | **N3** | AM087222 | avian |
| A/ostrich/Germany/R5-10/2006 (H5N3, LP) | **H5** | **N3** | not available | avian |
| A/mallard/Germany/R2557/2006 (H5N3) | **H5** | **N3** | not available | avian |
| A/mallard/Germany/R731/2008 (H5N3; LP) | **H5** | **N3** | not available | avian |
| A/mallard/Germany/R734/2008 (H5N3; LP) | **H5** | **N3** | not available | avian |
| A/mallard/Germany/R771/2008 (H5N3; LP) | **H5** | **N3** | not available | avian |
| A/mallard/Germany/R772/2008 (H5N3; LP) | **H5** | **N3** | not available | avian |
| A/turkey/Germany/R1550/2008 (H5N3; LP) | **H5** | **N3** | not available | avian |
| A/turkey/Germany/R1551/2008 (H5N3; LP) | **H5** | **N3** | not available | avian |
| A/turkey/Germany/R1557/2008 (H5N3; LP) | **H5** | **N3** | not available | avian |
| A/turkey/Germany/R1612/2008 (H5N3; LP) | **H5** | **N3** | not available | avian |
| A/Duck/Germany/R1789/2008 (H5N3; LP) | **H5** | **N3** | CY107849 | avian |
| A/turkey/Germany/R2379/2008 (H5N3; LP) | **H5** | **N3** | not available | avian |
| A/wild duck/Germany-BY/R2892/2009 (H5N3; LP) | **H5** | **N3** | EPI356413, EPI356412 | avian |
| A/duck/Potsdam/2216-4/1984 (H5N6; LP) | **H5** | **N6** | CY006036, CY005771 | avian |
| A/turkey/Ontario/7732/1966 (H5N9, HP) | **H5** | **N9** | AB558456, GU051967 | avian |
| A/mallard/British Columbia/544/2005 (H5N9) | **H5** | **N9** | not available | avian |
| A/mallard/Alberta/329/2006 (H5N9; LP) | **H5** | **N9** | KC790085, KC790087 | avian |
| A/turkey/Germany/R30/1999 (H6N1) | **H6** | **N1** | AJ507209 | avian |
| A/turkey/Germany/R81/1999 (H6N1) | **H6** | **N1** | not available | avian |
| A/turkey/Germany/R82/1999 (H6N1) | **H6** | **N1** | not available | avian |
| A/poultry/Germany/R3043/2007 (H6N1) | **H6** | **N1** | not available | avian |
| A/poultry/Germany/R3045/2007 (H6N1) | **H6** | **N1** | not available | avian |
| A/poultry/Germany/R3047/2007 (H6N1) | **H6** | **N1** | not available | avian |
| A/turkey/Gvulot Israel/2009 (H6N1) | **H6** | **N1** | not available | avian |
| A/turkey/Massachusetts/3740/1965 (H6N2) | **H6** | **N2** | CY087752, CY087754 | avian |
| A/turkey/Germany/R26/1999 (H6N2) | **H6** | **N2** | AJ507208 | avian |
| A/avian/Israel/289/2001 (H6N2) | **H6** | **N2** | JN564727, JN575028 | avian |
| A/avian/Israel/320/2001 (H6N2) | **H6** | **N2** | JN564729, JN575030 | avian |
| A/turkey/Germany/R04/2002 (H6N2) | **H6** | **N2** | not available | avian |
| A/turkey/Germany/R14/2002 (H6N2) | **H6** | **N2** | AJ507205 | avian |
| A/turkey/Germany/R25/2002 (H6N2) | **H6** | **N2** | not available | avian |
| A/turkey/Germany/R26/2002 (H6N2) | **H6** | **N2** | AJ507204 | avian |
| A/turkey/Germany/R30/2002 (H6N2) | **H6** | **N2** | not available | avian |
| A/turkey/Germany/R44/2002 (H6N2) | **H6** | **N2** | not available | avian |
| A/turkey/Germany/R45/2002 (H6N2) | **H6** | **N2** | not available | avian |
| A/turkey/Germany/R46/2002 (H6N2) | **H6** | **N2** | not available | avian |
| A/turkey/Germany/R48/2002 (H6N2) | **H6** | **N2** | not available | avian |
| A/turkey/Germany/R49/2002 (H6N2) | **H6** | **N2** | not available | avian |
| A/turkey/Germany/R54/2002 (H6N2) | **H6** | **N2** | not available | avian |
| A/turkey/Germany/R57/2002 (H6N2) | **H6** | **N2** | not available | avian |
| A/turkey/Germany/R58/2002 (H6N2) | **H6** | **N2** | not available | avian |
| A/turkey/Germany/R59/2002 (H6N2) | **H6** | **N2** | not available | avian |
| A/turkey/Germany/R60/2002 (H6N2) | **H6** | **N2** | not available | avian |
| A/turkey/Germany/R62/2002 (H6N2) | **H6** | **N2** | not available | avian |
| A/turkey/Germany/R63/2002 (H6N2) | **H6** | **N2** | not available | avian |
| A/turkey/Germany/R65/2002 (H6N2) | **H6** | **N2** | not available | avian |
| A/turkey/Germany/R617/2007 (H6N2) | **H6** | **N2** | EPI317612 | avian |
| A/duck/Germany/R2791/2007 (H6N2) | **H6** | **N2** | not available | avian |
| A/duck/Germany/R2793/2007 (H6N2) | **H6** | **N2** | not available | avian |
| A/duck/Germany/R2794/2007 (H6N2) | **H6** | **N2** | not available | avian |
| A/sentinel mallard/Germany/Sum57K/2007 (H6N2) | **H6** | **N2** | not available | avian |
| A/mallard/Germany/Sum62K/2007 (H6N2) | **H6** | **N2** | not available | avian |
| A/ringed teal/Germany/R641/2008 (H6N2) | **H6** | **N2** | not available | avian |
| A/duck/Germany/R1140/2008 (H6N2) | **H6** | **N2** | not available | avian |
| A/duck/ Germany/R1142/2008 (H6N2) | **H6** | **N2** | not available | avian |
| A/duck/Germany/R1143/2008 (H6N2) | **H6** | **N2** | not available | avian |
| A/duck/Germany/R1144/2008 (H6N2) | **H6** | **N2** | not available | avian |
| A/duck/Germany/R1149/2008 (H6N2) | **H6** | **N2** | not available | avian |
| A/duck/Germany/R1150/2008 (H6N2) | **H6** | **N2** | not available | avian |
| A/duck/Germany/R1151/2008 (H6N2) | **H6** | **N2** | not available | avian |
| A/mallard/Germany/R1501/2008 (H6N2) | **H6** | **N2** | not available | avian |
| A/greylag goose/Germany/R1625/2008 (H6N2) | **H6** | **N2** | not available | avian |
| A/mallard/Germany/R1710/2008 (H6N2) | **H6** | **N2** | not available | avian |
| A/mallard/Germany/R1711/2008 (H6N2) | **H6** | **N2** | not available | avian |
| A/wild goose/Germany-BB/R2329/2008 (H6N2) | **H6** | **N2** | EPI397608, EPI397607 | avian |
| A/mallard/Germany/Wv118/2005 (H6N4) | **H6** | **N4** | not available | avian |
| A/turkey/Germany/R43/1998 (H6N5) | **H6** | **N5** | AJ507206 | avian |
| A/wigeon/Germany/Wv579/2005 (H6N5) | **H6** | **N5** | not available | avian |
| A/mallard/Germany/sum156/2007 (H6N5) | **H6** | **N5** | EPI397610, EPI397609 | avian |
| A/turkey/Germany/R83/1999 (H6N8) | **H6** | **N8** | not available | avian |
| A/red-brested goose/Germany/R1/2006 (H6N8) | **H6** | **N8** | not available | avian |
| A/red-brested goose/Germany/R2/2006 (H6N8) | **H6** | **N8** | not available | avian |
| A/goose/Germany/R1122/2006 (H6N8) | **H6** | **N8** | not available | avian |
| A/ostrich/Germany/R1312/2006 (H6N8) | **H6** | **N8** | not available | avian |
| A/goose/Germany/R1767/2007 (H6N8) | **H6** | **N8** | EPI416259, EPI339174 | avian |
| A/mute swan/Germany/R2927/2007 (H6N8) | **H6** | **N8** | EPI185339, EPI185345 | avian |
| A/mute swan/Germany/R3231/2007 (H6N8) | **H6** | **N8** | not available | avian |
| A/ostrich/Germany/R48/2010 (H6N9) | **H6** | **N9** | not available | avian |
| A/FPV/Rostock/45/1934 (H7N1) | **H7** | **N1** | not available | avian |
| A/Alexandria tyrode/T145/1948 (H7N1) | **H7** | **N1** | not available | avian |
| A/chicken/Italy/444/1999 (H7N1; HP) | **H7** | **N1** | AJ704810 | avian |
| A/broiler/Italy/445/1999 (H7N1; HP) | **H7** | **N1** | CY107846 | avian |
| A/turkey/Italy/472/1999 (H7N1; LP) | **H7** | **N1** | AJ704811 | avian |
| A/chicken/Italy/473/1999 (H7N1; LP) | **H7** | **N1** | not available | avian |
| A/turkey/Ontario/18-1/2000 (H7N1; LP) | **H7** | **N1** | not available | avian |
| A/mallard/Germany/NVP41/2004 (H7N1; LP) | **H7** | **N1** | CY107853 | avian |
| A/teal/Föhr/Wv180/2005 (H7N2; LP) | **H7** | **N2** | not available | avian |
| A/chicken/Dgania/Israel/1980_R709/2009 (H7N2) | **H7** | **N2** | not available | avian |
| A/mallard/Alberta/49/1976 (H7N3; LP) | **H7** | **N3** | CY185713, CY185715 | avian |
| A/duck/Italy/636/2003 (H7N3) | **H7** | **N3** | not available | avian |
| A/turkey/Italy/2043/2003 (H7N3; LP) | **H7** | **N3** | CY022613, CY022615 | avian |
| A/chicken/British Columbia/CN-07/2004 (H7N3; HP) | **H7** | **N3** | EF470587 | avian |
| A/guineafowl/Germany/R2495/2007 (H7N3) | **H7** | **N3** | AM930528 | avian |
| A/sentinel-duck/Germany/SK207R/2007 (H7N3) | **H7** | **N3** | not available | avian |
| A/mallard/Germany/Sko212-219K/2007 (H7N3) | **H7** | **N3** | not available | avian |
| A/swan/Germany/R736/2006 (H7N4; LP) | **H7** | **N4** | EPI492517 | avian |
| A/mallard/Germany/R756/2006 (H7N4) | **H7** | **N4** | not available | avian |
| A/chicken/Brescia/1902 (H7N7; HP) | **H7** | **N7** | GU186777, GU186779 | avian |
| A/chicken/Leipzig/1979 (H7N7; HP) | **H7** | **N7** | U20459 | avian |
| A/duck/Potsdam/15/1980 (H7N7; LP) | **H7** | **N7** | CY081275, CY081277 | avian |
| A/duck/Potsdam/13/1980 (H7N7; LP) | **H7** | **N7** | not available | avian |
| A/swan/Germany/62/1981 (H7N7; LP) | **H7** | **N7** | AJ704798 | avian |
| A/turkey/Ireland/PV8/1995 (H7N7; LP) | **H7** | **N7** | CY107857 KF160885 | avian |
| A/chicken/Germany/R28/2003 (H7N7; HP) | **H7** | **N7** | AJ620350, AJ620349 | avian |
| A/mallard/GermanyWv190/2005 (H7N7) | **H7** | **N7** | not available | avian |
| A/swan/Germany/R57/2006 (H7N7; LP) | **H7** | **N7** | EPI492518 | avian |
| A/mallard/Germany/R721/2006 (H7N7) | **H7** | **N7** | not available | avian |
| A/greylag goose/Germany/R752/2006 (H7N7) | **H7** | **N7** | not available | avian |
| A/mallard/Alberta/8734/2007 (H7N7, LP) | **H7** | **N7** | AM933238 | avian |
| A/mallard/Germany/R192/2009 (H7N7) | **H7** | **N7** | not available | avian |
| A/turkey/Germany-NW/R655/2009 (H7N7; LP) | **H7** | **N7** | EPI356351, EPI356352 | avian |
| A/avian/ / R224/2010 | **H7** | **N7** | not available | avian |
| A/equi/Prague/1/1956 (H7N7) | **H7** | **N7** | X62552 | equine |
| A/Anser spec./Germany/R44/2006 (H8N3) | **H8** | **N3** | AM922158 | avian |
| A/turkey/Ontario/6118/1968 (H8N4) | **H8** | **N4** | CY014659, CY130048 | avian |
| A/mallard/Germany/R2167/2009 (H8N4) | **H8** | **N4** | not available | avian |
| A/turkey/Wisconsin/1/1966 (H9N2) | **H9** | **N2** | CY130054, CY130056 | avian |
| A/chicken/Pakistan/AG519/1998 (H9N2) | **H9** | **N2** | AJ781823 | avian |
| A/chicken/United Arab Emirates/AG537/1999 (H9N2) | **H9** | **N2** | AJ781824 | avian |
| A/chicken/Israel/90658/2000 (H9N2) | **H9** | **N2** | EF492221, EF492301 | avian |
| A/turkey/Israel/90710/2000 (H9N2) | **H9** | **N2** | AY738451, EF492273 | avian |
| A/chicken/Saudi Arabia/AG516/2000 (H9N2) | **H9** | **N2** | AJ781826 | avian |
| A/chicken/Iran/AG541/2000 (H9N2) | **H9** | **N2** | AJ781825 | avian |
| A/chicken/China/AG518/2001 (H9N2) | **H9** | **N2** | AJ781827 | avian |
| A/chicken/Emirates/R66/2002 (H9N2) | **H9** | **N2** | not available | avian |
| A/chicken/Iran/R64/2002 (H9N2) | **H9** | **N2** | not available | avian |
| A/chicken/Saudi-Arabia/R61/2002 (H9N2) | **H9** | **N2** | not available | avian |
| A/turkey/Israel/965/2002 (H9N2) | **H9** | **N2** | AY738452, EF492289 | avian |
| A/turkey/Israel/1209/2003 (H9N2) | **H9** | **N2** | EF492238, EF492291 | avian |
| A/ostrich/Israel/1436/2003 (H9N2) | **H9** | **N2** | AY738456, EF492294 | avian |
| A/turkey/Israel/1567/2004 (H9N2) | **H9** | **N2** | EF492241, EF492297 | avian |
| A/chicken/Israel/29/2005 (H9N2) | **H9** | **N2** | EF492222, EF492274 | avian |
| A/falcon/Dubai/R2063/2006 (H9N2) | **H9** | **N2** | not available | avian |
| A/chicken/Israel/178/2006 (H9N2) | **H9** | **N2** | EF492224, EF492276 | avian |
| A/chicken/Israel/1525/2006 (H9N2) | **H9** | **N2** | FJ46472, FJ464627 | avian |
| A/chicken/Israel/215/2007 (H9N2) | **H9** | **N2** | FJ464716, FJ464615 | avian |
| A/chicken/Israel/386/2007 (H9N2) | **H9** | **N2** | FJ464720, FJ464619 | avian |
| A/turkey/Israel/900/2007 (H9N2) | **H9** | **N2** | FJ464725, FJ464624 | avian |
| A/avian/Germany/R220/2008 (H9N2) | **H9** | **N2** | not available | avian |
| A/avian/Germany/R221/2008 (H9N2) | **H9** | **N2** | not available | avian |
| A/avian/Germany/R222/2008 (H9N2) | **H9** | **N2** | not available | avian |
| A/avian/Germany/R226/2008 (H9N2) | **H9** | **N2** | not available | avian |
| A/avian/Germany/R249/2008 (H9N2) | **H9** | **N2** | not available | avian |
| A/avian/Germany/R250/2008 (H9N2) | **H9** | **N2** | not available | avian |
| A/avian/Germany/R251/2008 (H9N2) | **H9** | **N2** | not available | avian |
| A/chicken/Israel/292/2008 (H9N2) | **H9** | **N2** | FJ464717, FJ464616 | avian |
| A/chicken/Israel/524/2008 (H9N2) | **H9** | **N2** | FJ464723, FJ464622 | avian |
| A/avian/Israel/824/2005 (H10N2) | **H10** | **N2** | JN564733, JN575034 | avian |
| A/mallard/Föhr/Wv1298-1302/2003 (H10N4) | **H10** | **N4** | not available | avian |
| A/mallard/Germany/Wv1677-81/2003 (H10N4) | **H10** | **N4** | AM922160 | avian |
| A/mallard/Germany/1682-85/2003 (H10N4) | **H10** | **N4** | not available | avian |
| A/moorhen/Germany/Wv1703/2004 (H10N4) | **H10** | **N4** | not available | avian |
| A/common buzzard/Germany/R1184/2006 (H10N4) | **H10** | **N4** | not available | avian |
| A/mute swan/Germany/R2201/2006 | **H10** | **N4** | not available | avian |
| A/chicken/Germany/N/1949 (H10N7) | **H10** | **N7** | GQ176136, GQ176134 | avian |
| A/avian/Israel/232/2001 (H10N7) | **H10** | **N7** | JN564726, JN575027 | avian |
| A/avian/Israel/445/2001 (H10N7) | **H10** | **N7** | JN564730, JN575031 | avian |
| A/Mallard/Germany/Wv1722/2003 (H10N7) | **H10** | **N7** | not available | avian |
| A/Mallard/Germany/1776-80/2003 (H10N7) | **H10** | **N7** | not available | avian |
| A/Mallard/Föhr/Wv1781-82/2003 (H10N7) | **H10** | **N7** | not available | avian |
| A/mallard/Germany/Wv886/2004 (H10N7) | **H10** | **N7** | not available | avian |
| A/mallard/Germany/Wv906/2004 (H10N7) | **H10** | **N7** | not available | avian |
| A/mallard/Germany/Wv907/2004 (H10N7) | **H10** | **N7** | not available | avian |
| A/mallard/Germany/Wv941/2004 (H10N7) | **H10** | **N7** | not available | avian |
| A/mallard/Germany/Wv1014/2004 (H10N7) | **H10** | **N7** | not available | avian |
| A/Mallard/Germany/Wv1015/2004 (H10N7) | **H10** | **N7** | not available | avian |
| A/mallard/Germany/1506/2006 (H10N7) | **H10** | **N7** | not available | avian |
| A/mallard/Germany/2074/2007 (H10N7) | **H10** | **N7** | not available | avian |
| A/mallard/Germany/2075/2007 (H10N7) | **H10** | **N7** | EPI317614 | avian |
| A/avian/Israel/543/2008 (H10N7) | **H10** | **N7** | JN564732, JN575033 | avian |
| A/mallard/Germany/1488/2009 (H10N7) | **H10** | **N7** | not available | avian |
| A/mallard/Germany/1490/2009 (H10N7) | **H10** | **N7** | not available | avian |
| A/mallard/Germany/1501/2009 (H10N7) | **H10** | **N7** | not available | avian |
| A/mallard/Germany/1502/2009 (H10N7) | **H10** | **N7** | not available | avian |
| A/mallard/Germany/1505/2009 (H10N7) | **H10** | **N7** | not available | avian |
| A/Eurasian Coot /Germany/411/2010 (H10N8) | **H10** | **N8** | KJ508885 | avian |
| A/coot/Germany/1128/2006 (H11N1) | **H11** | **N1** | not available | avian |
| A/Domestic Duck/Germany/R784/2006 (H11N1) | **H11** | **N1** | EPI341615 | avian |
| A/waterfowl/Germany/R350/2008 | **H11** | **N1** | not available | avian |
| A/avian/Israel/2001_R715/09 (H11N2) | **H11** | **N2** | not available | avian |
| A/duck/England/1956 (H11N6) | **H11** | **N6** | CY130062, CY130064 | avian |
| A/?/Germany/R2795/2006 (H11N6) | **H11** | **N6** | not available | avian |
| A/mallard/Germany/Wv1499-1503/2003 (H11N9) | **H11** | **N9** | not available | avian |
| A/Anas platyrhynchos/Germany/R2219/2006 (H11N9) | **H11** | **N9** | AM922161 | avian |
| A/wigeon/Germany/R636/2007 (H11N9) | **H11** | **N9** | not available | avian |
| A/mallard/Germany/R3108/2007 (H11N9) | **H11** | **N9** | not available | avian |
| A/duck/Germany/R3349/2009 (H11N9) | **H11** | **N9** | not available | avian |
| A/duck/Alberta/60/1976 (H12N5) | **H12** | **N5** | CY130078, CY130080 | avian |
| A/Lesser black-backed gull/Finland/R2195/2009 (H13N2) | **H13** | **N2** | not available | avian |
| A/Lesser black-backed gull/ Finland/R2263-69/2009 (H13N2) | **H13** | **N2** | not available | avian |
| A/Lesser black-backed gull/Finland/R2265/2009 (H13N2) | **H13** | **N2** | not available | avian |
| A/pilot whale/Maine/328/84 (H13N2) | **H13** | **N2** | KJ372720, KJ372722 | mammalian |
| A/gull/Maryland/704/1977 (H13N6) | **H13** | **N6** | CY130086, CY130088 | avian |
| A/gull/Stralsund/Wv1136-40/2003 | **H13** | **N6** | AM922163 | avian |
| A/jackdaw/Germany/WV1141KR/2003 (H13N6) | **H13** | **N6** | AM087220 | avian |
| A/Bird/Germany/R1642/2007 (H13N6) | **H13** | **N6** | not available | avian |
| A/Larus ridibundus/Germany/R2064/2006 (H13N8) | **H13** | **N8** | AM922164 | avian |
| A/Black headed gull/Germany/R2622/06 (H13N8) | **H13** | **N8** | not available | avian |
| A/mallard/Gurjev/263/1982 (H14N5) | **H14** | **N5** | AM922165 | avian |
| A/shearwater/Australia/2576/1979 (H15N9) | **H15** | **N9** | CY130102, CY130104 | avian |
| A/black-headed gull/Sweden/5/99 (H16N3) | **H16** | **N3** | AY684891, AY684905 | avian |
| A/herring gull/Germany-MV/R2788/2006 (H16N3) | **H16** | **N3** | EPI356422, EPI432616 | avian |
| A/herring gull/Germany-MV/R2792/2006 (H16N3) | **H16** | **N3** | EPI356417, EPI356419 | avian |
| A/herring gull/Germany/R3309/2007 (H16N3) | **H16** | **N3** | not available | avian |

Table S2: Diagnostic validation of RITA results from IAV-positive avian and porcine swab samples; detailed overview.

| **Sample no.** | **Sample origin** | **RITA results** | | **Sequencing results** | |
| --- | --- | --- | --- | --- | --- |
|  |  | **specific** | **non-specific^#^** | **pan-HA** | **pan-NA** |
| AR 1836-14 | wild bird | H3N8 | H10 | H3 | N8 |
| AR 1840-14 | wild bird | H3N4 | H10, N8 | - | - |
| AR 1841-14 | wild bird | H3N8 | H10 | H3 | N8 |
| AR 1875-14 | wild bird | H12N6 | - | - | N6 |
| AR 2303-14 | wild bird | H5N8 | - | H5 | N8 |
| AR 2305-14 | wild bird | H5N8 | - | - | N8 |
| AR 2316-14 | wild bird | H5N8 | - | H5 | N8 |
| AR 2476-14 | wild bird | H6(N2)* | - | H6 | N2 |
| AR 2478-14 | wild bird | H6(N2) | - | - | N2 |
| AR 2788-14 | wild bird | H6N1 | - | - | N1 |
| AR 3208-14 | wild bird | H6N1 | - | H6 | N1 |
| AR 3235-14 | wild bird | H5N3 | - | H5 | N3 |
| AR 3282-14 | wild bird | H3, H5, H6, H9, N2, N8 | H10, (H5) | - | N2 |
| AR 3288-14 | wild bird | H4N6 | - | H4 | N6 |
| AR 3290-14 | wild bird | H8N1 | H4 | H8 | N1 |
| AR 3291-14 | wild bird | H8 | H4 | H8 | N1 |
| AR 3116 | wild bird | H11N9 | - | - | N9 |
| AR 3130 | wild bird | H1N1 | - | H1 | N1 |
| AR 3155 | wild bird | (H1)N1 | - | H1 | N1 |
| AR 3515 | wild bird | H11N9 | - | H11 | N9 |
| AR 3537 | wild bird | H9N2 | - | H9 | N2 |
| AR 3542 | wild bird | (H16)N3 | - | H16 | N3 |
| AR 1535-15-2 | wild bird | H13N8 | - | - | N8 |
| AR 2199-15 | wild bird | H11N9 | - | H11 | N9 |
| AR 2218-15 | wild bird | H3, H6, N2, N8 | - | - | N8 |
| AR 2220-15 | wild bird | H3, H6, N8 | H10 | H6 | - |
| AR 2225-15 | wild bird | H6N8 | H3 | - | - |
| AR 2288-15-2 | wild bird | H12N5 | - | - | - |
| AR 2327-15 | wild bird | H6N2 | - | - | - |
| AR 2431-15 | wild bird | H11N9 | - | H11 | N9 |
| AR 2451-15-2 | wild bird | H1N1 | - | H1 | N1 |
| AR 2454-15-2 | wild bird | H2N3 | - | H2 | N3 |
| AR 2455-15-2 | wild bird | H2N3 | - | H2 | N3 |
| AR 2458-15-2 | wild bird | H3N8 | H10 | H3 | N8 |
| AR 2459-15-2 | wild bird | H3N6 | H10 | H3 | N6 |
| AR 2460-15-2 | wild bird | H3N6, N4 | H10 | H3 | N6 |
| AR 2464-15-2 | wild bird | H11N9 | - | H11 | N9 |
| AR 2466-15-2 | wild bird | H5N2 | - | H5 | N2 |
| AR 2823-15 | wild bird | H5N3 | (H11) | H5 | N3 |
| AR 2825-15 | wild bird | H11N3 | (H5) | - | N3 |
| AR 2911-15-1 | wild bird | H5N3 | - | H5 | N3 |
| AR 3231-15-2 | wild bird | H5N2 | H2, H7 | H5 | N2 |
| AR 3232-15-2 | wild bird | H5N2 | H7 | H5 | N2 |
| AR 3264-15-2 | wild bird | H5N2 | - | H5 | N2 |
| AR 3289-15-2 | wild bird | H6N2 | H1 | H6 | N2 |
| AR 547-15 | swine | H3N2 | H10 | H3 | - |
| AR 650-15 | swine | H1N1 | - | H1 | N1 |
| AR 680-15 | swine | H1N2 | - | H1 | N2 |
| AR 856-15 | swine | H1N2 | - | H1 | N2 |
| AR 1052-15 | swine | H1N1 | - | H1 | N1 |
| AR 1105-15 | swine | H1N1 | - | H1 | N1 |
| AR 1148-15 | swine | H1N1 | - | H1 | N1 |
| AR 1203-15 | swine | H3N2 | - | H3 | N2 |
| AR 1355-15 | swine | H1N2 | - | H1 | N2 |
| AR 1843-15 | swine | H1N1 | - | H1 | N1 |
| AR 1996-15 | swine | H1N2 | - | H1 | N2 |
| AR 2061-15 | swine | H1N1 | - | H1 | N1 |
| AR 2367-15 | swine | H1N1 | - | H1 | N1 |
| AR 2379-15 | swine | H1N1 | - | H1 | N1 |
| AR 2383-15 | swine | H1N1, N2 | - | H1 | N1 |
| AR 2413-15 | swine | H1N2 | - | H1 | N2 |
| AR 2618-15 | swine | H1N1 | - | H1 | N1 |

Figure S1:


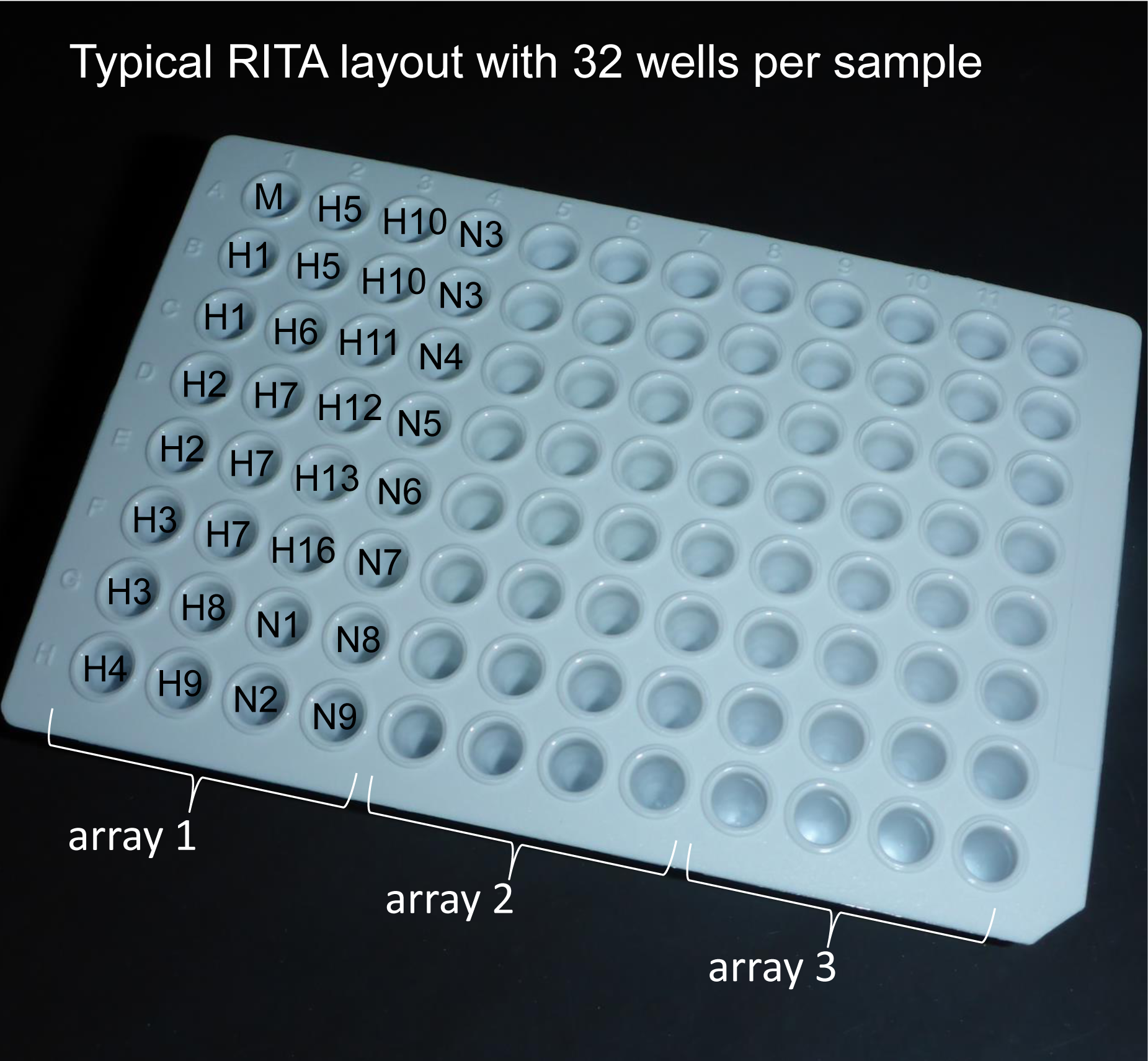

Supplement: Supplementary Information [file srep27211-s1.doc]
